# DAPTEV: Deep aptamer evolutionary modelling for COVID-19 drug design

**DOI:** 10.1371/journal.pcbi.1010774

**Published:** 2023-07-05

**Authors:** Cameron Andress, Kalli Kappel, Marcus Elbert Villena, Miroslava Cuperlovic-Culf, Hongbin Yan, Yifeng Li

**Affiliations:** 1 Department of Computer Science, Brock University, St. Catharines, Canada; 2 Broad Institute of MIT and Harvard, Cambridge, Massachusetts, United States of America; 3 Department of Biological Sciences, Brock University, St. Catharines, Canada; 4 Digital Technologies Research Centre, National Research Council Canada, Ottawa, Canada; 5 Department of Chemistry, Brock University, St. Catharines, Canada; University of Maryland School of Pharmacy, UNITED STATES

## Abstract

Typical drug discovery and development processes are costly, time consuming and often biased by expert opinion. Aptamers are short, single-stranded oligonucleotides (RNA/DNA) that bind to target proteins and other types of biomolecules. Compared with small-molecule drugs, aptamers can bind to their targets with high affinity (binding strength) and specificity (uniquely interacting with the target only). The conventional development process for aptamers utilizes a manual process known as Systematic Evolution of Ligands by Exponential Enrichment (SELEX), which is costly, slow, dependent on library choice and often produces aptamers that are not optimized. To address these challenges, in this research, we create an intelligent approach, named DAPTEV, for generating and evolving aptamer sequences to support aptamer-based drug discovery and development. Using the COVID-19 spike protein as a target, our computational results suggest that DAPTEV is able to produce structurally complex aptamers with strong binding affinities.

## Author summary

Compared with small-molecule drugs, aptamer drugs are short RNAs/DNAs that can specifically bind to targets with high strength. With the interest of discovering novel aptamer drugs as an alternative to address the long-lasting COVID-19 pandemic, in this research, we developed an artificial intelligence (AI) framework for the in silico design of novel aptamer drugs that can prevent the SARS-CoV-2 virus from entering human cells. Our research is valuable as we explore a novel approach for the treatment of SARS-CoV-2 infection and the AI framework could be applied to address future health crises.

## Introduction

Viruses contain *deoxyribonucleic acid* (DNA) or *ribonucleic acid* (RNA) but are incapable of self-reproduction and rely on commandeering the cell’s protein creation capabilities to reproduce. After the viral protein has been successfully reproduced, it goes on to infect other cells in a process known as *viral proliferation*. Attaching and injecting of viral DNA or RNA to host cells is achieved through binding to cellular receptor through *receptor-binding domain* (RBD), an area on the viral protein evolved to specifically bind hosts’ cell receptor. In the case of the SARS-CoV-2 spike protein, the RBD targets the lung cell angiotensin-converting enzyme (ACE2) receptor [1–6].

Typical drug discovery focuses on either preventative triggering of host’s immune system or interrupting the life cycle of the virus. The former usually refers to vaccines. The latter is known as a therapeutic, attempting to halt the infection process in a currently-infected host [1, 7, 8]. While vaccines tend to focus on infection prevention [8, 9], therapy development can help alleviate suffering following infection.

*Aptamers* are short, single-stranded, oligonucleotides that can bind to targets with high affinity and specificity. It is hypothesized that specific aptamers can halt a virus during its life cycle by binding to the viral protein RBD thereby inhibiting its binding to host cell receptors. Aptamers can be created and modified easily and can bind to specific targets. One of the main ways to design aptamers is through a process known as *systematic evolution of ligands by exponential enrichment* (SELEX) [10] or *high-throughput SELEX* (HT-SELEX) [11] which applies high-throughput sequencing in each SELEX cycle. Design of aptamers through either SELEX or HT-SELEX is a slow, highly experimentally demanding process, possibly still providing sub-optimal designs [12–14] as these approaches essentially rely on a random search of top hits that are highly dependent on the initial choice of libraries [1, 8, 14–20].

*Artificial intelligence* (AI) techniques involve the modelling of brain intelligence to solve various challenging tasks [21]. As the most influential area in AI, *machine learning* (ML) improves intelligent agents or models using data and experience [22]. In the past few decades, machine learning has seen unprecedented progress. A variety of supervised and generative *deep learning* (DL) methods, *i.e*. neural networks, have been designed and achieved state-of-the-art performances in many domains [23]. Even though various AI and ML models have been recently developed for small-molecule drug design and achieved promising results [24–27], the end-to-end design of aptamers is a relatively unexplored domain [16, 19]. This is partially due to the unfamiliarity of aptamers in the AI community, unavailability of quality data, and the shortage of chemoinformatics tools for this class of molecules. In this research, to determine if AI approaches can accelerate aptamer drug discovery and development for the treatment of SARS-CoV-2 infection, we developed an *in silico* RNA aptamer design process, named *deep aptamer evolutionary model* (DAPTEV), similar to the experimental SELEX process. DAPTEV takes advantage of the embedding and generating capacity of *deep generative models* (DGMs), the high-throughput exploration power of evolutionary computation, and the quantitative measure of RNA-target binding affinities through molecular mechanism. The performance of DAPTEV was evaluated using the SARS-CoV-2 spike protein as a target. This work has the following major contributions: (1) we designed a novel end-to-end aptamer generation and optimization model to explore its performance, which is highly informative to researchers in the drug design domain; (2) using the SARS-CoV-2 spike protein as a target, we assembled a benchmark dataset for aptamer drug development, which can be used and improved by researchers in the future; (3) we applied our framework to search for RNA aptamer therapeutics for COVID-19 treatment, discovering new aptamers that can be further validated as an alternative means to address our pandemic challenge.

## Related work

Until recently, patents for the SELEX process limited the potential exploration and innovation in the field of aptamer-based drug development [19]. Moreover, sources on ML applied to aptamers were even more scarce. However, with the patents now expired, new research is being released (reviewed as below), some of which implement a model related to the one proposed in this research.

### Unsupervised aptamer identification methods

A naive method for aptamer selection in SELEX is based on read counts. However, due to bias in *polymerase chain reaction* (PCR) [28], the high abundance of reads does not indicate a high binding affinity. To address this issue, early computational methods focused on aptamer identification from SELEX pools using *clustering* and *motif finding*. Clustering techniques, such as AptaCluster [29] and FASTAptamer-Cluster [30], attempt to learn aptamer sequence commonalities, but suffer from a lack of secondary structure considerations. Motif-finding approaches, such as MEMRIS [31], Aptamotif [32], MPBind [28], and APtaTrace [33], attempt to identify structure patterns with strong binding affinities. However, motif-finding approaches do not consider entire secondary structures. Both options struggle with processing times associated to large HT-SELEX libraries. To address these issues while utilizing the strengths of these two approaches, *Sequential Multidimensional Analysis AlgoRiThm for aptamer discovery* (SMART-Aptamer) is presented in [14] which accurately and efficiently identifies aptamers from SELEX libraries containing hundreds of millions of short sequences. SMART-Aptamer first applies a Markov clustering method to obtain aptamer families and then filters out the majority of the aptamers using multiple scores considering the enrichment of motifs, the abundance of aptamer families, and the overall secondary structures.

### Supervised aptamer-protein interaction prediction methods

Aptamers that bind to a small number of protein targets have been selected to form limited aptamer-protein interaction (API) data, enabling the use of supervised models to learn from known APIs and then predict whether a new aptamer interacts with one of the protein targets listed in the training data. Due to limited training samples, conventional classifiers and hand-crafted features have been applied to API prediction and obtained moderate performance (70–80% accuracy). In [34] a *random forest* classifier learns on an API dataset containing a few hundred positive samples. Their input features include nucleotide composition, traditional amino acid composition, and pseudo amino acid composition. Similarly, in [35], an ensemble method (random forests) with hybrid features is presented to predict APIs using *Pseudo K-tuple Nucleotide Composition* (PseKNC) features to encode aptamers along with protein features including *discrete cosine transformation* (DCT), disorder information, and bi-gram *position specific scoring matrix* (PSSM). The task of ncRNA-protein interaction prediction is similar to API prediction, but has more data available to enable the training of a deep classifier. For instance in [36], RPITER, a hierarchical deep learning model with *convolutional neural network* (CNN) and *autoencoder* (AE) modules, is developed to automatically learn features from a few thousands ncRNA-protein pairs and obtained promising performance for ncRNA-protein interaction prediction. When more API data becomes available in the future, similar deep models are anticipated for API prediction.

### Aptamer generation

Im *et al*. used a generative model to build statically sized (20 nt) DNA and RNA sequences that bind to a target protein [37]. At the time of their publication, it was specified that their research was ongoing, but they were able to train a *long short-term memory* (LSTM) model on a “huge dataset of sequences from high-throughput experimental technologies … such as HT-SELEX or CLIP-seq”. This dataset, which is still not available publicly, was obtained from DeepBind [38]. DeepBind was used to estimate the affinity and specificity of generated sequences. The target proteins in their research were as follows: DRGX, GCM1, OLIG1, RXRB, NFATC1, NFKB1, and MBNL1. It was found that the produced sequences possessed structural motifs similar to known motifs, and that the produced sequences had a strong binding affinity and specificity to their intended target [37]. This research was continued by Park *et al*. and a similar conclusion was drawn for RNA aptamers specifically [39]. In [40], Iwano *et al*. propose RaptGen which is similar to [37] and [39]. They too utilized DeepBind, however, RaptGen implemented a CNN-LSTM as their encoder and a profile *hidden Markov model* as their decoder. Additionally, while RaptGen starts with static sequence lengths (10 nt), it utilizes some post-processing techniques to extend the sequence lengths to a set of other fixed sizes, technically achieving sequence generation with variable lengths. Iwano *et al*.’s target proteins were the TGM2 and *α*v*β*3 [40]. The main difference between the research mentioned above and our own is as follows: (1) our model allows for the specification of a target RBD and calculating binding affinity based on thermodynamic principles rather than estimating binding via ML surrogate models; (2) our model optimizes aptamers to produce sequences with better binding affinity and specificity to the intended target instead of only generating aptamers having the same distribution as the training data; and (3) our model allows for true variable length sequences as input and output rather than fixed sized sequences.

In [13], Lee *et al*. present an aptamer generation method called Apt-MCTS. This method uses limited positive and negative aptamer-protein pairs to train a random forest classifier as a surrogate model to predict whether an aptamer can bind to a fixed protein target (one of 6GOF, 3V79_1, 5VOE, 3SN6_4, 2RH1_1, and 1ERK_1). The prediction result is used as a score to guide a *Monte Carlo tree search* (MCTS) [41] process for the generation of new aptamers. Apt-MCTS and our DAPTEV research utilize the same starting real aptamer data, but we further expanded this data by generating additional RNA sequences for model training. Apt-MCTS requires the availability of aptamer-protein pairs to train a surrogate model for API predictions. This disables the application of Apt-MCTS in our situation where experimental aptamer-spike protein interaction data are unavailable.

Wornow proposed a conditional VAE to generate novel strong-binding aptamers [19]. This too is similar to our work and findings. However, Wornow’s work utilized 8 rounds of SELEX data as a proof of concept on DNA data only. This creates a limitation of requiring experimental data to be collected before the model’s efficacy can be illustrated or be used in practice and does not explore the RNA landscape. The target for Wornow’s research is the chemotherapeutic agent daunomycin. Binding affinity was approximated based on ground truth fitness scores which were later confirmed by wet-lab experiments. They avoid the use of *molecular dynamics* (MD) simulations, stating they are computationally expensive and infeasible for use in the field.

### Molecular optimization

Grantham *et al*. have developed a multi-objective deep learning framework named *deep evolutionary learning* (DEL) for small-molecule design. DEL is beneficial for improving sample populations in terms of property distributions and outperforms other multi-objective baseline molecular optimization algorithms [26]. While DEL does not work with aptamer data, it does focus on molecular optimization. Thus, it serves as inspiration for our DAPTEV.

## Method

**Fig 1:**
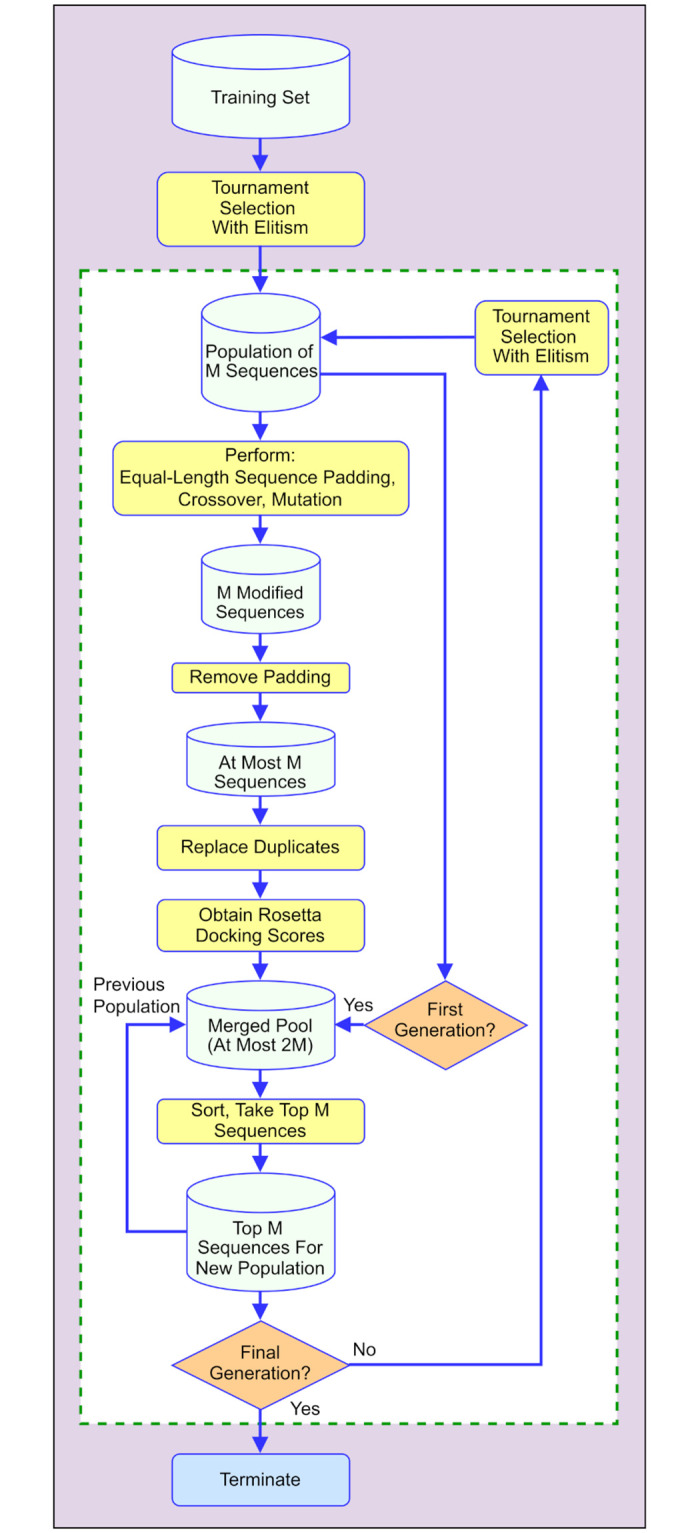
Flowchart depicting the DAPTEV process for aptamer design.

Our *deep aptamer evolutionary modelling* (DAPTEV) framework is visualized in Fig 1. It is a hybrid approach that integrates the strengths of DGMs (variational autoencoders) for aptamer encoding and modelling, computational intelligence (evolutionary computation) for aptamer optimization, and bioinformatics tools for RNA secondary and tertiary structure prediction and RNA-protein folding-and-docking (Rosetta). The continuous docking score is used as the fitness value or objective in the evolutionary computation component to guide aptamer optimization.

First, a dataset must be collected for the training of the deep learning model and the initialization of the optimization process. The dataset should include a large number of aptamer sequences of certain lengths, their corresponding secondary structures, and docking scores to a protein target of interest. The secondary structures (determined by canonical base-pairing formed by hydrogen bonds) are needed by the Rosetta package [42] for docking. If secondary structures are unknown, they can be quickly computed using Arnie [43]. For details of assembling data to generate aptamers targeting SARS-CoV-2 spike protein, please refer to Section Data.

As an initialization step, a *variational autoencoder* (VAE) [44] is pretrained using the dataset. This VAE provides an encoding process that transforms an aptamer sequence to a vector of continuous values which is called the *embedding vector* or *latent vector* of the aptamer. Since the latent space is continuous, various metaheuristic techniques can be applied in this space for aptamer optimization. The decoder of the VAE enables a decoding process that converts a latent vector (either randomly sampled or modified using computational intelligence operations) to an aptamer sequence. Please refer to Section Variational autoencoder for aptamer modelling for details of our developed VAE.

The main body of the DAPTEV framework is an iterative evolutionary process. At the beginning of the loop, a population of aptamers are randomly sampled from the original dataset, then they are passed to the encoder to obtain latent representations. Evolutionary operations are applied to these latent representations and then these modified latent representations are decoded using the decoder to obtain their corresponding primary sequences. The fitness of these new sequences can be measured through aptamer-protein docking. After that, the newly produced aptamer sequences and the previous population of aptamer sequences are ranked together and only top aptamers are kept to form a new population of aptamers for the next generation. In the next generation, this new population is used to fine-tune the VAE model and then uses the encoder of the fine-tuned VAE to project the new population into the latent space where evolutionary operations are again applied. See Section Evolutionary operations for aptamer optimization for detailed discussion of the evolutionary operations. Each time when a new aptamer sequence is generated, Rosetta needs to be called to calculate the docking score. The docking component is detailed in Section RNA-protein docking. Furthermore, docking is a time-consuming process. Its speed-up is discussed in Section Computing time improvement.

### Variational autoencoder for aptamer modelling

**Fig 2:**
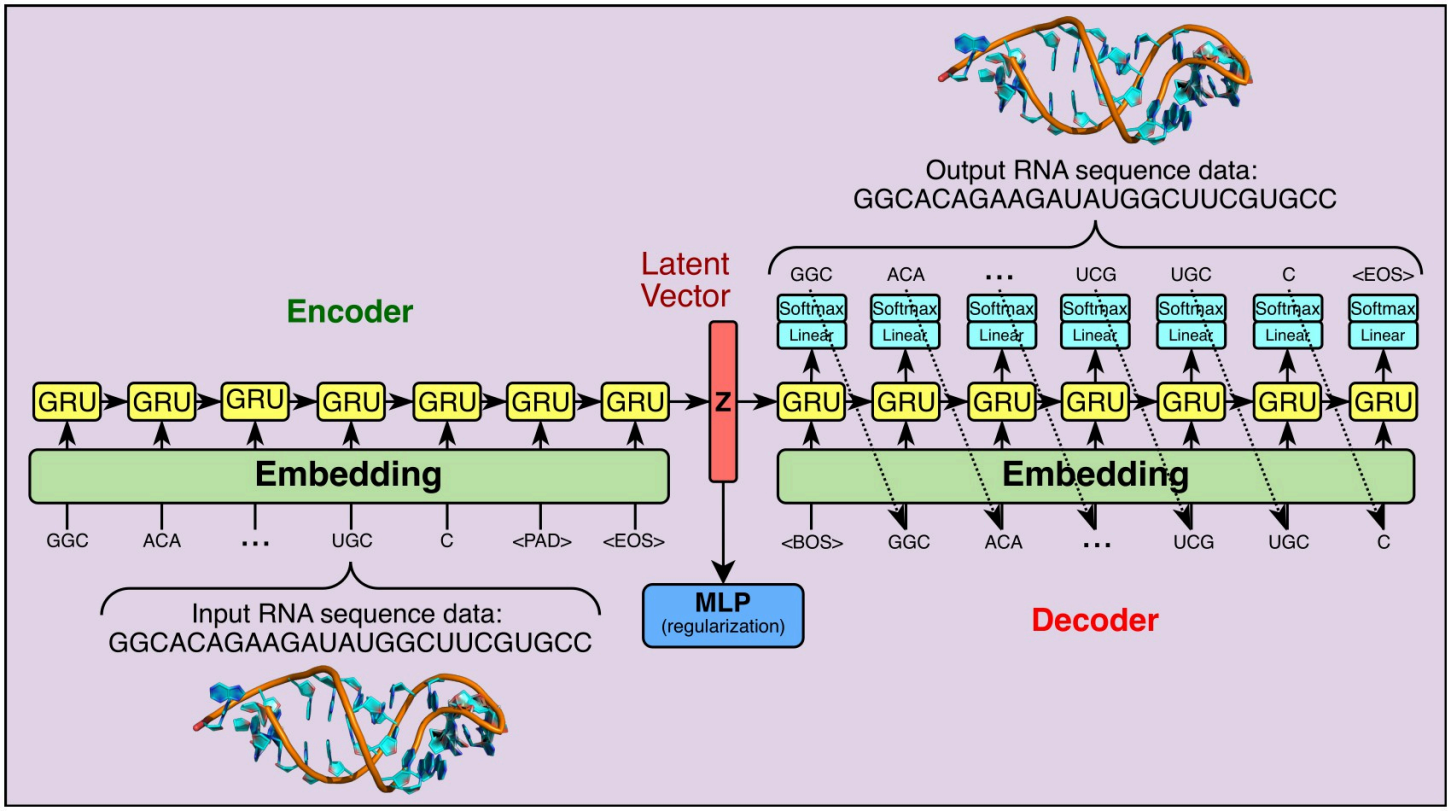
Depiction of the implemented VAE.

VAE is a probabilistic deep generative model that takes advantage of the autoencoder architecture where the encoder network corresponds to the inference *q*_***ϕ***_(***z***|***x***) and the decoder network realizes the generative component *p*_***θ***_(***z***)*p*_***θ***_(***x***|***z***). Here ***x*** is the vector of random variables for observed data (i.e., an aptamer sequence in our case), ***z*** is the vector of latent random variables, ***ϕ*** and ***θ*** are respectively the parameters of the inference network (encoder) and generative network (decoder) [44]. In our work, since aptamers are represented in primary sequences, *gated recurrent units* (GRU) [45] are used in the encoding and decoding processes. Thus, the resulted VAE is a probabilistic Seq2Sep structure. See Fig 2 for a depiction of the implemented VAE.

Using “A” for adenine, “C” for cytosine, “G” for guanine, and “U” for uracil, an aptamer sequence of length *L* can be represented as ***x*** = [*x*_*i*_|*x*_*i*_ ∈ {*A*, *C*, *G*, *U*}, *i* = 1, 2, ⋯, *L*]. The *word embedding* representation techniques [46, 47] can be used here by treating each letter as a word. However, this alone does not provide the model with enough context for how an RNA structure will interact with itself and the possible structural motifs that can occur. Thus, the combination of all possible 1, 2, and 3-mer strings (as tokens) are included in the vocabulary V={A,C,G,U,AA,AC,⋯,UU,AAA,AAC,⋯,UUU}. Conversely, adding too many combinations could drastically increase the computing time and the need for more training data. Thus, we restrict our vocabulary up to 3-mer. Three more special tokens are inserted into the vocabulary: <BOS> for beginning of sequence, <EOS> for end of sequence, and <PAD> for padding. Both the <BOS> and the <EOS> tokens inform DAPTEV of the character boundaries for each sequence. When working with sequences, one inevitably must choose how to handle sequences of variable lengths. Rather than restricting all sequences to the same length, limiting the versatility and learning capabilities of the model, <PAD> tokens are inserted into each sequence. In the encoding process, the sequence needs to be parsed into tokens. The 3-mer tokens have the highest priority in this process. A 2 or 1-mer token only appears at the end of the parsed result when a 3-mer parsing is no longer possible. Taking sequence GGCACAGAAGAUAUGGCUUCGUGCC for example, the parsed sequence of tokens is [GGC, ACA, GAA, GAU, AUG, GCU, UCG, UGC, C]. The decoding process follows a similar scheme. The decoding will stop when (1) it generates a 2 or 1-mer token, (2) an <EOS> token is generated, or (3) the length of the generated sequence meets the prespecified maximal length.

The bottleneck between the encoder and decoder is the latent layer representing the embedding of the whole sequence. From the autoencoder perspective, a latent vector contains the compressed key information about the corresponding input and this latent vector can be decoded to reconstruct the input. From the generative model perspective, the latent vector, following a simple prior distribution (a multivariate standard Gaussian distribution in our case), is the start of the generation process. The feasible domain of the latent vector forms the latent space.

Up to this point, the VAE designed above is not necessarily learning what makes an RNA sequence good or bad with respect to the Rosetta docking score. The VAE should be able to produce sequences with strong docking capabilities to a specified target. It is for this reason that an MLP component was included in the architecture of the VAE. This MLP performs score-based classification and further regularizes the latent representation. It takes a latent vector as input and predicts whether the sequence associated with the latent vector is good in terms of target binding, this vector and its associated score will be given to the MLP. A lower docking score implies a tighter binding. Thus, a “good docking score” is defined as ≤ the user-chosen score threshold parameter and a “bad docking score” is defined as > the score threshold. This converts the scores to 1 (good) or 0 (bad). Note that one cannot simply set the score threshold to any low value desired as it can result in highly imbalanced data. To determine the optimal score threshold, one may locate the lowest score that still labels at least 25% of the data to Class 1 (as a rule of thumb only). For example, it was found that 26.28% of our data (see Section Data) fell within the docking score range of 3,500 and below for this research. Thus, a score threshold of 3,500 was chosen in our experiment. Why is the docking quality prediction modelled as a classification problem rather than a regression problem? According to our experience in docking score modelling, regression usually has unsatisfactory performance.

The loss function of the VAE to be minimized in DAPTEV is formulated below

$$\begin{array}{cc} & l\left(\phi ,\theta ,\psi \right)=-{\text{E}}_{q_{\phi }\left(z|x\right)}\left[\text{log}\;p_{\theta }\left(x|z\right)\right]+\beta \text{KL}(q_{\phi }\left(z|x\right)\left|\right|p_{\theta }\left(z\right))\\ & +\alpha {\text{E}}_{q_{\phi }\left(z|x\right)}[\text{BCE}(f_{\psi }\left(z\right),y)], \end{array}$$ (1)

where the first term corresponds to the reconstruction error, the second term is the *Kullback-Leibler* (KL) divergence that pushes the posterior towards the prior, and the last term is the *binary cross-entropy* (BCE) loss for the MLP (parameterized by ***ψ***) performance where *f*_***ψ***_(***z***) is the predicted value and *y* is the actual class label. The trade-off hyperparameters *α* and *β* control the balance among these three terms. The KL loss term tends to vanish easily during training in the vanilla VAE where *β* = 1. This can negatively affect the reconstruction learning process. To address this issue, we implemented a technique known as *KL annealing* [26, 48] to control the value of *β*. The annealing process applies a small weight, between 0 and 1, to the KL loss value and linearly increases this weight during training.

### Evolutionary operations for aptamer optimization

Each evolutionary generation ensures the VAE has finished a round of training (fine-tuning) such that DAPTEV can then begin the sequence optimization step. This is when DAPTEV searches for better sequences and update its running data accordingly in every generation. Three Darwinian evolutionary operations are involved in this step.

The first operation is known as *tournament selection with elitism*. *Elitism* is the action of selecting the top *e* best individuals (sequences) in a population to be carried forward into the next generation. This ensures the best individuals remain in the running data and are not filtered out during tournament selection. Tournament selection is the process of randomly sampling sequences from the population and having these candidates compete in a tournament. The winner of each tournament will be selected as a *parent* to produce the next generation of *children*. This process is repeated multiple times until the parent pool equals the chosen population size. The tournament selection operation is controlled by two hyperparameters. One is the *k* value which indicates how many contenders with which to build a tournament round. The higher the *k* value, the more likely a stronger performing individual will be selected to win the tournament and proceed as a parent for the next generation. This, in turn, means there is a higher likelihood that poor-performing individuals will not be selected to proceed as parents (referred to as *selection pressure*), which can often have a detrimental effect on exploration. The other hyperparameter is the *selection rate*. This parameter creates a possibility for the best-performing individual in a tournament pool to opt out, allowing some poor-performing individuals to be selected instead.

The second evolutionary operation is *crossover*. This is the process of producing children from two parents, attempting to simulate a child obtaining features from their parents. After the selection operation is performed, the sequences are passed through the VAE’s encoder to obtain their latent representations (***z***s). Then, the crossover operation is applied to these parent ***z*** vectors whereby parent ***z***_*pa*_ and parent ***z***_*pb*_ will each copy random parts of their latent vectors to new children vectors ***z***_*ca*_ and ***z***_*cb*_. This procedure is repeated until the number of children equals the population size. Similar to the tournament selection, this operation also has a probability of occurring (called *crossover rate*). Note that too small of a population size will result in a low amount of “*genetic diversity*” which can quickly converge to a local minimum (suboptimal solution). This means that there will not be enough sequences in a given generation to produce sufficiently dissimilar children from the parent sequences. Eventually, all produced sequences could start to resemble each other and/or the algorithm will be unable to find better sequences due to the lack of “genetic diversity” among available sequences.

The last evolutionary operation is *mutation*, mimicking random mutations in evolution. Some random influence should be allowed such that an individual may find themselves performing significantly better than the rest. In DAPTEV, mutation is implemented by selecting, with a probability called *mutation rate*, a child from the crossover result. Then a random index of that child’s ***z*** vector is chosen and replaced with a random, normally distributed value. The mutation rate should be very small. A large rate will result in the algorithm essentially performing just a random search, corrupting any learning.

Once the new latent vectors have completed a round of evolutionary operations, they are sent through the VAE’s decoder. This will generate the sequences based on the VAE’s previously trained decoding capabilities. The output will be a list of new sequences. Any duplicates in this list, as compared to themselves, the starting data, and any previously predicted sequences will be removed and replaced with new, folded, random sequences. However, docking scores for these new sequences are unknown. Thus, the docking simulation is repeated before the VAE continues to fine-tune using the new population.

### RNA-protein docking

Different from previous sequential prefold-and-dock methods, Rosetta’s *RNP-denovo* [42] models RNA-protein complexes by simultaneously folding and docking an RNA to a protein surface via a statistical RNA-protein docking scoring function [42]. It is reported in [42] that, over ten popular scoring systems, Rosetta was the most accurate among the methods tested. It is for this reason that Rosetta was used as a docking platform in this research. Meanwhile, the readers are reminded that, highly precise RNA-protein docking remains a challenging problem in the community partially due to the lack of high-resolution RNA-protein complexes. While Rosetta’s method tends to correctly determine global folds, it is not accurate enough to reliably determine atomic positions. Further improvement of Rosetta for RNA-protein docking is necessary in the future, but beyond the scope of this paper.

Rosetta allows one to specify a target protein and the target’s RBD. There are two scoring functions within DAPTEV’s usage of Rosetta: *native Rosetta docking score function* and *constraint scoring function*. The native Rosetta scoring function is a low-resolution, coarse-grained, knowledge-based (statistical) RNA-protein potential. This serves as an energy function for scoring Monte Carlo steps within the tertiary structure prediction and docking simulation. The lower the returned score is after the tertiary structure prediction and docking simulation, the more stable the predicted complex is considered to be. In Rosetta, all previously published score terms describing RNA structure and RNA-protein interactions are included in this scoring function [42], while also providing rapid computation and maintaining coarse-granularity.

The constraint scoring function applies to where on the protein the RNA docks. This constraint function allows one to specify the target’s RBD region without having to force a fixed binary interaction between specific atoms of the RNA and the target protein. Instead, one indicates to Rosetta their chosen constraint type, to which atoms on the target and the RNA the constraint applies, and what built-in formula the constraint will use to calculate an energetic penalty. This penalty will be applied against the returned Rosetta score to passively discourage the RNA from docking elsewhere on the target. As a result, the RNA has a range of acceptable distances it can deviate from the target’s RBD during the docking simulation.

DAPTEV uses an “atom pair” constraint with a flat harmonic function. The middle position (nucleotide) of every produced RNA sequence is chosen as the constrained RNA nucleotide. This function is formulated below,

$$\begin{array}{c} f\left(x\right)=\{\begin{array}{cc} 0 & \text{if}\;\;x_{0}-tol\leq x\leq x_{0}+tol\\ (\frac{x-x_{0}}{sd}{)}^{2} & \text{otherwise} \end{array}, \end{array}$$ (2)

where, *x* represents the distance between the two atoms (this varies as Rosetta attempts multiple conformations), *x*_0_ is the user-chosen ideal distance between the atoms, *sd* is the user-chosen allowable standard deviation from the ideal distance, and *f*(*x*) is the returned penalty. This function produces a penalty of 0 if *x* falls in the range [*x*_0_ − *tol*, *x*_0_ + *tol*], where *tol* means tolerance. All units of distance are measured in Ångströms. In our research, we set *x*_0_ = 0, *sd* = 0.125, and *tol* = 1.

### Computing time improvement

A challenge for utilizing Rosetta in DAPTEV is the computational demand and time required to perform docking simulations. For an experiment with population size 800, generation number 10, and 3 runs, it could take about 4 years if the *full* SARS-CoV-2 spike protein was used and docking simulations ran *sequentially*. The running time can be reduced to roughly one-third of a year by reducing the protein file to just the RBD. However, this is still too long to wait for one experiment to finish. As such, *multiprocessing* was implemented into DAPTEV. Multiprocessing is a technique to run multiple docking simulations on a computer’s multiple CPUs at the same time (in parallel). DAPTEV utilized 18 CPUs to effectively reduce computing time for each docking simulation from roughly 6.5 minutes (also using the reduced protein) to 1 minute per aptamer sequence.

## Experimental results

### Data

**Fig 3:**
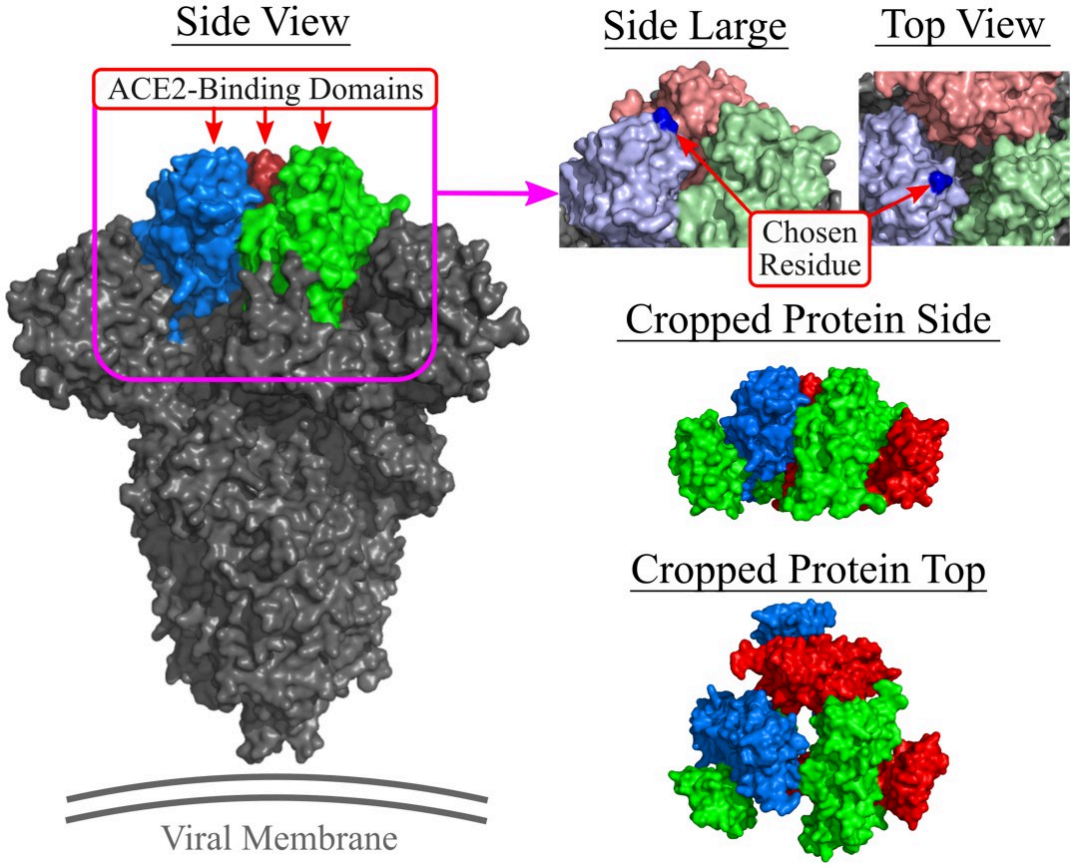
Visualizing the spike protein and the cropped version. Location of the chosen residue (201A, atom C5, dark blue dot) on the renumbered SARS-CoV-2 spike protein RBD. Note: the full spike protein shown (left) is for reference but the protein file in DAPTEV is cropped. PDB: 6VXX.

In our experiments, we used the SARS-CoV-2 spike glycoprotein (closed state, PDB: 6VXX) [5] as the docking target with an RBD between residues 333 and 524 [2, 4–6, 49]. See Fig 3 for an illustration. As it shows, the SARS-CoV-2 spike protein’s RBD represents a relatively small area at the apical face of the protein. Using the entire spike protein in the docking procedure would drastically increase the computation time. For example, performing five runs of the docking process with an RNA base count of 25 on the full SARS-CoV-2 spike protein took over 98 minutes to finish computing. Thus, one should remove all unnecessary residues from the target PDB file and save the new PDB structure and the new FASTA sequence. Unnecessary residues are those on the target that an RNA could not possibly interact with during the RBD-docking process due to constraint specification. Once these residues are removed, the PDB file must be cleaned (renumbered and sequenced).

For the implementation in the docking, the protein is cropped according to the RBD and renumbered. The chosen RBD residue number is 201 (499 in the original pdb file) and the associated chain is “A” for the Rosetta constraint. Fig 3 shows a visual of the chosen residue. The carbon atom “C5” is ubiquitous among all four standard RNA bases [50]. This was the reason behind choosing “C5” as the RNA atom parameter. Doing so required no additional calculations to be performed and was simple to implement.

While an entire dataset can be created using the random sequence generator, it is best to augment the dataset with known aptamers. Providing existing aptamer data will add diversity into the dataset, will expose the model to physical characteristics of existing aptamers, and will help the model learn these traits. This will also allow DAPTEV to rule out or confirm existing aptamers as potential solutions to the given problem as it is possible that a known aptamer is already a good fit for the specified target. As a result, supplying existing aptamer data could provide DAPTEV with a strong starting position before it begins exploring other options.

We do not allow unfolded RNA secondary structures to be created when performing random sequence generation. Associated secondary structures will be restricted to having at least one set of brackets (base pair connections). It has been observed during the experimental phase that unfolded secondary structures produce RNA tertiary structures that are more malleable when docking to the target’s RBD. Thus, unfolded sequences can better form to the target’s RBD topology, producing strong binding scores (binding affinity). However, aptamers are designed not only for high binding affinity but also for target specificity [1, 8, 15–18, 20]. An unfolded structure would not be considered “specific” to a target because it could potentially bind just as well to another target. Furthermore, the Rosetta scoring function for the docking process includes the quality of the RNA tertiary structure prediction. This means that the returned score for any sequence in this model may be deceptively “improved” due to encountering few stability-related penalties during the structure prediction and docking process. As a result, the scores produced by unfolded RNA structures are artificially and significantly better than their more structurally-complex peers. This can result in the algorithm producing many unfolded RNA aptamers, which would not be ideal for aptamer drug development as the goal is to optimize the aptamer discovery process for a specific target only. While binding specificity is a challenging requirement to evaluate [51], in this paper we instead assess fold rate which is a weaker criterion on structural complexity.

After obtaining 849 unique known aptamer sequences [13, 52, 53], only 390 met the condition of being between the minimum (20) and maximum (40) sequence lengths. Of those 390 aptamer, 344 contained at least one connection (base pairing) in their secondary structures. 44 sequences were too small, 417 sequences were too large and an additional 44 sequences were unfolded (based on the returned Arnie secondary structure predictions). As 12,000 data points were required, an additional 11,656 random sequences were generated and scored. These sequences were restricted to having lengths between 20 and 40 nts, an approximate GC percentage of 50%, and containing at least one secondary structure connection.

### Hyperparameter setting

In our experiments, the settings of hyperparameters for the data collection process, the evolutionary optimization process, and the structure and training of the VAE are listed in Table 1.

**Table 1:** DAPTEV hyperparameters used in our experiments.

Initial Data Collection Hyperparameters			
**Min Seq Length**	20	**Max Seq Length**	40
**GC Content**	50%	**Force Folded**	True
**Data Size**	12,000		
Evolutionary Optimization Hyperparameters			
*k* **in Tournament**	2	**Selection Rate**	0.95
**Elitism Rate**	0.01	**Crossover Rate**	0.01
**Population Size**	800	**# Generations**	10
**Repeated Runs**	3		
Encoder Structure			
**Embedding Size**	87	**Hidden Layers**	[256]
*μ* **Layer**	[512]	log *σ* **Layer**	[512]
**Latent Vector Size**	10	**Bidirectional**	True
Decoder Structure			
**Hidden Layers**	[512,1024]	**Output Layer**	[87]
**Bidirectional**	False		
MLP Structure			
**Hidden Layers**	[64,32]	**Output Layer**	[1]
VAE-Specific Training Hyperparameters			
**Score Threshold**	3,500	**Pretrain. Epochs**	45
**Fine-tun. Epochs**	10	**Initial Learn. Rate**	3e-3
**End Learn. Rate**	3e-4	**Starting** *β*	1e-3
**End** *β*	0.5	*α*	1

### Baselines

The experiment of DAPTEV is accompanied by three additional comparison models: the DAPTEV model with the starting 344 real aptamer data points replaced by randomly generated sequences containing at least one base pairing in each secondary structure (DAPTEV-X), a genetic algorithm on the sequence space (GA), and a “hill climber” algorithm (HC).

DAPTEV-X was provided to illustrate the difference in performance when running the DAPTEV model with real aptamer data and excluding this data. It is possible that adding only 344 real aptamer data points will be insignificant when compared to the 12,000 total data points utilized in this experiment. However, it is also possible that adding even a few real aptamers will introduce the DAPTEV model to some real-world motifs, structures, and other patterns that the random sequence generator cannot capture. While the main focus of this research is to create a generative model that simulates and expedites the SELEX process, comparing the model’s performance with and without real data can provide scientists with more information on the effectiveness of the model and how best to utilize it. Nonetheless, it is important to include these real aptamers in the model such that they can be explored as potential drug candidates for the given target.

The GA baseline is to simply remove the VAE from DAPTEV, resulting in a GA operating on the problem space rather than the latent space, to see how their performance differs. It is worth noting that the VAE and the GA are not restricted to producing only folded structures. As such, these models do have a possibility of generating unfolded sequences. This is allowed to more deeply explore the solution landscape and potentially encounter stronger sequences through the prediction process. For the crossover function in GA, instead of selecting random indices of parent ***z***_*pa*_ and parent ***z***_*pb*_ (latent vectors), parents ***x***_*pa*_ and ***x***_*pb*_ will have random indices of the sequence space selected for crossover. The resulting children will be two new sequences. For the mutation function in GA, an individual is selected with a small probability. If selected, a random index of within the sequence is randomly chosen such that the corresponding letter is replaced with a random token from the vocabulary list excluding <BOS>, <PAD>, and <EOS>.

The HC baseline generates *random* sequences using our dataset creation script with the same chosen hyperparameters as the DAPTEV. This process is repeated for the same number of sequences, generations, and runs as the DAPTEV. The best performing sequences are carried forward into the next generation and all other sequences up to the chosen population size are discarded. This is known as a “hill climber” because this algorithm employs the simple heuristic of “always take the best” without any actual “learning”.

### VAE performance

**Fig 4:**
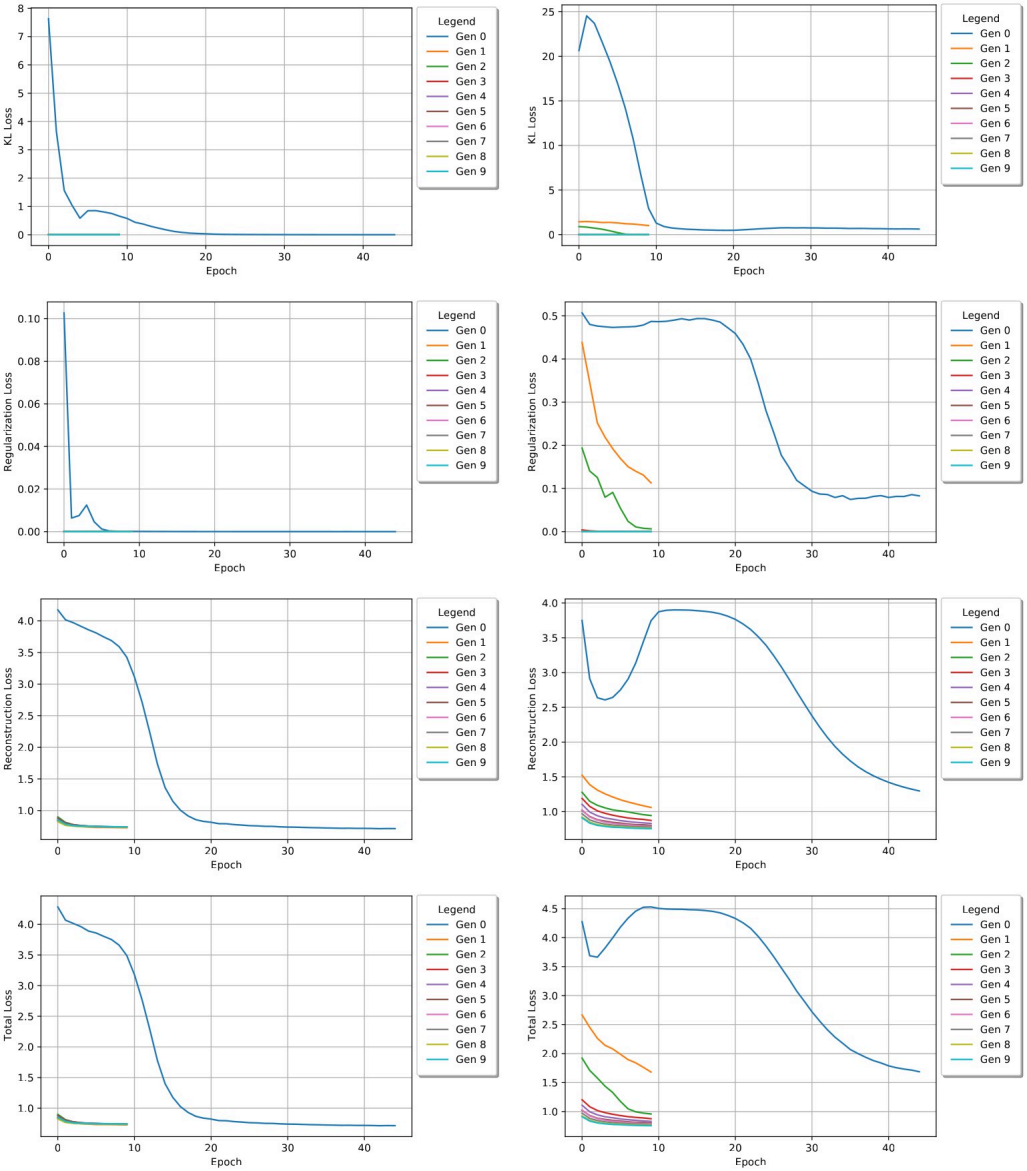
DAPTEV and DAPTEV-X comparison. KL loss, MLP regularization loss, reconstruction loss, and total loss values (top to bottom respectively) per epoch for each generation. The left graphs show the VAE performance with the starting 344 real aptamer data points. The right graphs show the performance of the VAE after replacing the 344 data points with randomly generated sequences (DAPTEV-X). Note: total loss values include the KL weight factor over time.

Fig 4 shows how the VAE’s encoder, MLP, and decoder modules performed per epoch and over each generation (plotted separately on the same graph). The total loss values are also shown in this figure. In the first generation, the VAE is trained on the entire dataset of 12,000 sequences. It shows that the KL loss can quickly decrease to a small value after 10 epochs. Each subsequent generation shows the encoder module continues to improve on the KL loss and approaches a near zero loss value. The MLP regularization module performs similarly. It shows that this module is also refining its performance. In fact, the module learned to perfectly classify the data halfway through generation five. The reconstruction loss graph suggests that, after the first generation, the decoder is learning how to reproduce the encoded latent vectors very well. The total loss graph shows the VAE’s overall performance per epoch per generation. As each model effectively reduced its loss values, it stands to reason that the total loss would reflect this performance. The right side of Fig 4 shows all the same information, but for the DAPTEV-X comparison model. Here, we can see that removing the 344 real aptamer data points has significantly affected the performance of each module’s ability to learn. The KL loss started significantly higher, became worse in epoch 1 before beginning to converge, and did not converge until the third generation. The regularization module performs much worse and does not converge until the fourth generation. It also starts significantly higher. The reconstruction module starts in a similar position to the DAPTEV model, but clearly has difficulty learning as quickly as DAPTEV did. The total loss is still quite similar to the reconstruction module’s performance, but it is clearly affected by both the regularization and KL modules more than the DAPTEV model is. Thus, the presence of the real aptamer data points did help the DAPTEV model learn better than the DAPTEV-X variant.

### Comparison in terms of docking score

Table 2 provides details on the overall docking score optimization performance for each model. Here, it can be seen that both DAPTEV and the GA improved significantly from their worst scores to their best scores, with GA outperforming DAPTEV. However, in comparison to the hill climber model, both DAPTEV and GA have performed better. One can also observe that DAPTEV performed better than DAPTEV-X in every docking score metric.

**Table 2:** The average score performance in the first and last generations for each experiment. Standard deviation provided in brackets. DAPTEV, DAPTEV-X, and GA were, respectively, run 3 times. HC was simulated based on our original dataset, thus was only run once. DAPTEV-X represents the DAPTEV model with the starting 344 real aptamer data points replaced with random sequences prior to running the simulation.

Docking Score		DAPTEV	DAPTEV-X	GA	HC
**First Gen**	*Best*	118 (14)	128 (0)	100 (41)	18,250
	*Mean*	897 (207)	1032 (7)	781 (337)	23,635
	*Median*	925 (204)	1056 (9)	801 (343)	21,957
	*Worst*	1,256 (297)	1457 (3)	1,098 (489)	60,677
**Last Gen**	*Best*	72 (31)	90 (29)	32 (24)	128
	*Mean*	578 (49)	603 (18)	292 (58)	1,098
	*Median*	602 (49)	634 (15)	303 (59)	1,122
	*Worst*	796 (77)	844 (21)	390 (86)	1,530

**Fig 5:**
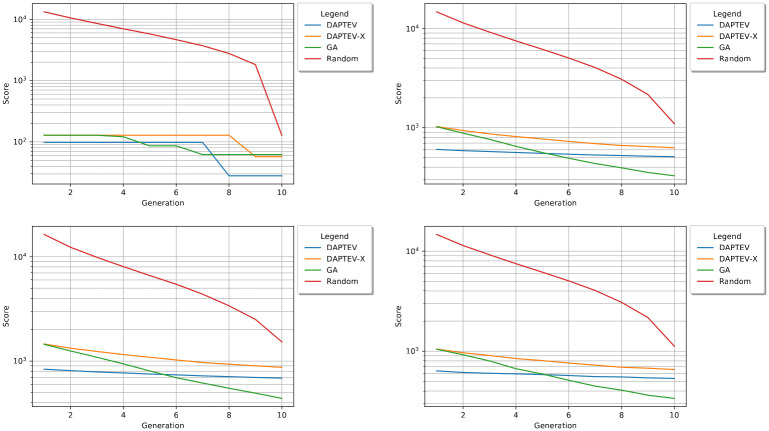
Best, worst, mean, and median score performance. The best (top-left), worst (bottom-left), mean (top-right), and median (bottom-right) scores plotted per generation on a logarithmic scale in a computational run for all three models. Note: DAPTEV-X refers to the DAPTEV model, but with the starting 344 real aptamer data points replaced with randomly generated sequences.

Fig 5 shows the best, worst, mean, and median performing docking scores produced by each model per generation. The provided graphs are scaled logarithmically as the hill climber (“Random” in the legend) experiment performed significantly worse than DAPTEV, DAPTEV-X, and GA. It is clear that GA produced better results than DAPTEV in terms of docking scores. The lowest (best) score produced by DAPTEV was 98 in generation three, whereas GA obtained its lowest score of 4 in generation nine. DAPTEV’s worst scores fell from 1,470 to 857 whereas GA’s worst score fell from 1,439 to 460. Both the means and medians seem to follow a fairly similar trend and are comparable in score output. Additionally, in every situation, the DAPTEV model outperformed DAPTEV-X.

**Fig 6:**
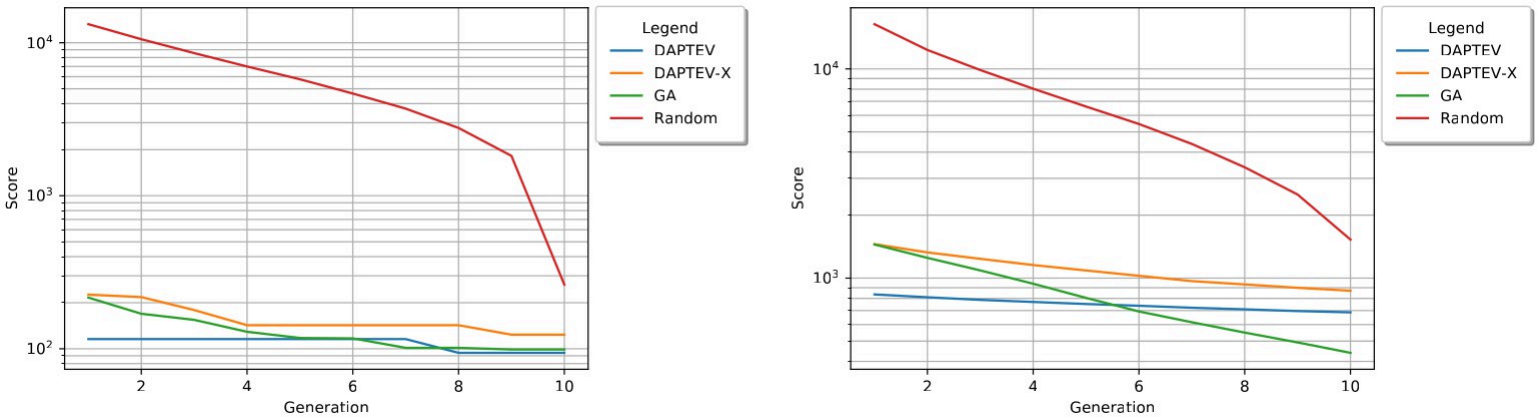
Best and worst 5 mean scores’ performance. The three models’ 5 best (left) and worst (right) mean scores plotted per generation on a logarithmic scale. DAPTEV-X, the DAPTEV model with the 344 starting aptamers replaced with random sequences, is also included here for comparison.

It also seemed prudent to consider the best five and worst five mean scores for each experiment. The absolute best and worst can send an extreme message and may not convey the true performance of each experiment. This is because the absolute best and worst do not include neighbouring sequence performance. If these models are indeed performing well, then one would expect to see the sequences near the best and worst improving overall. Fig 6 depicts the best and worst five score means per generation. Here, one can see that GA is indeed performing better than DAPTEV, and DAPTEV better than DAPTEV-X, in terms of docking score statistics across generations.

**Fig 7:**
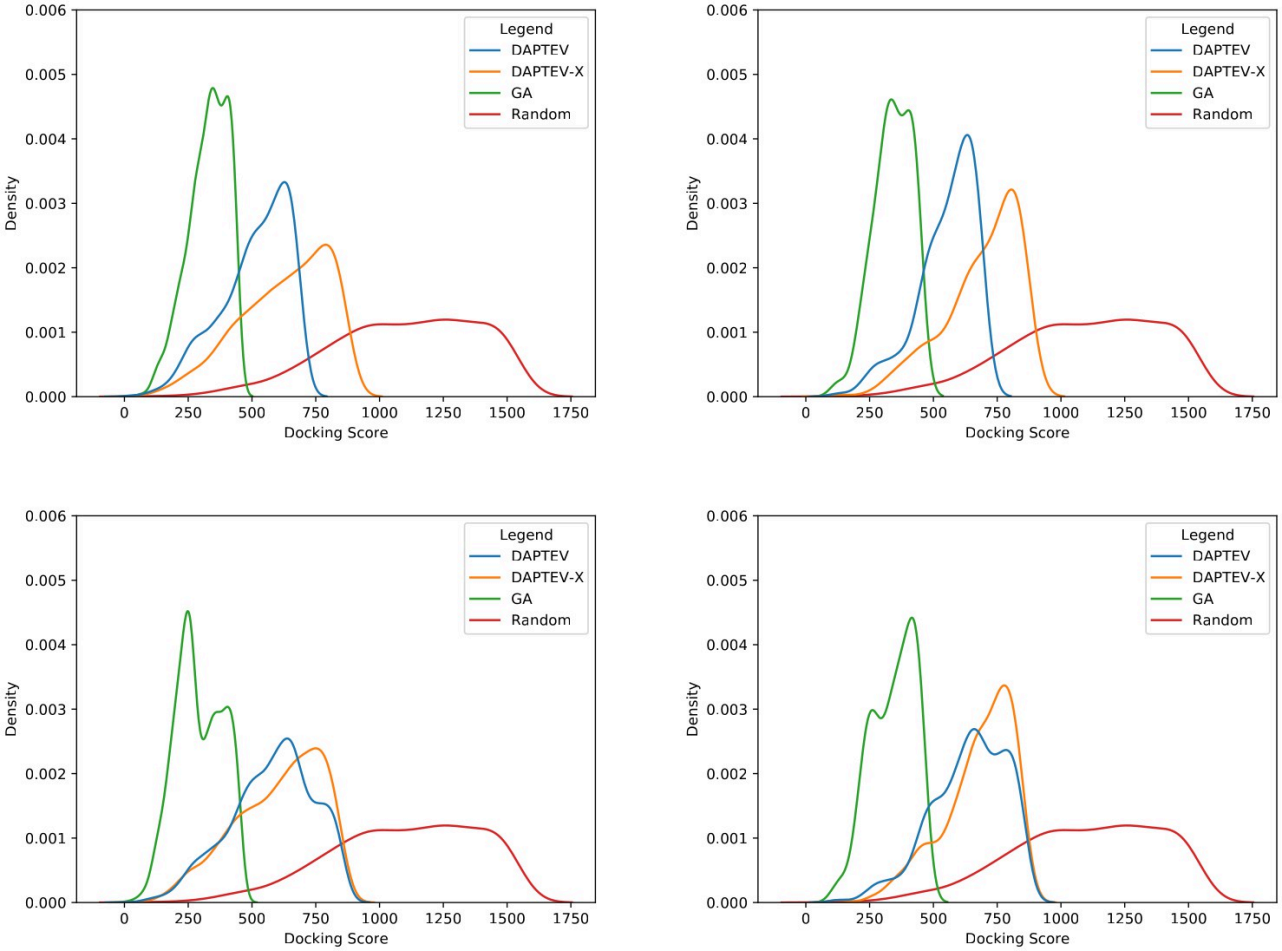
Density curves of binding scores. Density curves of binding scores for aptamer samples in the last generations of DAPTEV, GA, and HC (Random). DAPTEV-X refers to the DAPTEV model’s performance when the 344 starting aptamer data points are replaced with random sequences. The top-left graph shows all scores produced in each model’s best run. The top-right graph shows all folded complex scores from each model’s best run. The bottom-left shows all scores from all three runs. The bottom-right shows all folded complex scores from all three runs.

Furthermore, to have a full view of the binding score distribution for each method, the density curves of the aptamers’ binding scores in the last generations of DAPTEV, DAPTEV-X, GA, and HC (i.e. Random) are displayed in Fig 7. From the density plots, one can conclude that (1) DAPTEV, DAPTEV-X, and GA obtained aptamer sequences with significantly better binding scores than HC (Mann-Whitney U test: p-value = 7.25e-226, p-value = 3.15e-181, and p-value = 2.31e-256, respectively), (2) providing real aptamer data positively impacts the DAPTEV model’s performance, and (3) GA’s result is better than DAPTEV’s result in terms of docking score distribution (Mann-Whitney U test, p-value = 8.47e-142).

### Comparison in terms of fold rate

Thus far, it has seemed as though GA is the best suited model for this application. However, there are two goals for this experiment. While the first goal is to optimize the docking scores, the second goal is to produce sequences with at least one connection (nucleotide base pairing) in the secondary structures. In other words, these models were also supposed to learn the key features of well-performing structural motifs applied to the SARS-CoV-2 spike protein RBD.

Table 3 provides further details on the produced folded sequences. GA performed rather poorly in this task, only producing 47 (on average) folded structures out of 800 total sequences, achieving an 6% fold rate. Conversely, DAPTEV produced 272 (on average) folded structures out of 800 total sequences, achieving a 34% fold rate. While DAPTEV-X produced marginally more folded RNAs than DAPTEV, combining this knowledge with the previous graphs and tables indicates that these folded sequences from DAPTEV-X have lower docking scores. This can be observed by the amount of skew present for DAPTEV-X in Fig 7. Furthermore, each docking metric in Table 3 also shows DAPTEV outperforming DAPTEV-X. It is important to note that the HC (Random) algorithm is entirely constructed of folded RNAs as the dataset creation script does not allow for unfolded structures in its random search.

**Table 3:** Average docking scores and base pairings relating to the folded RNAs in each model. Standard deviation provided in brackets. Note: all values relate only to the folded RNAs in the last generation.

		DAPTEV	DAPTEV-X	GA	HC
	*Folded*	272 (35)	286 (19)	47 (21)	100
	*Rate (%)*	34 (4)	36 (2)	6 (3)	100
**Docking Scores**	*Best*	128 (0)	128 (0)	128 (0)	128
	*Mean*	631 (57)	674 (12)	315 (62)	1,098
	*Median*	664 (57)	706 (15)	324 (65)	1,122
	*Worst*	796 (77)	844 (21)	388 (88)	1,530
**Base Pairs**	*Minimum*	1.0 (0)	1.0 (0)	1.0 (0)	1.0
	*Mean*	2.3 (0.1)	2.3 (0)	2.1 (0.1)	2.6
	*Median*	2.0 (0)	2.0 (0)	2.0 (0)	3.0
	*Maximum*	5.0 (0)	5.0 (0)	3.7 (0.5)	7.0

**Fig 8:**
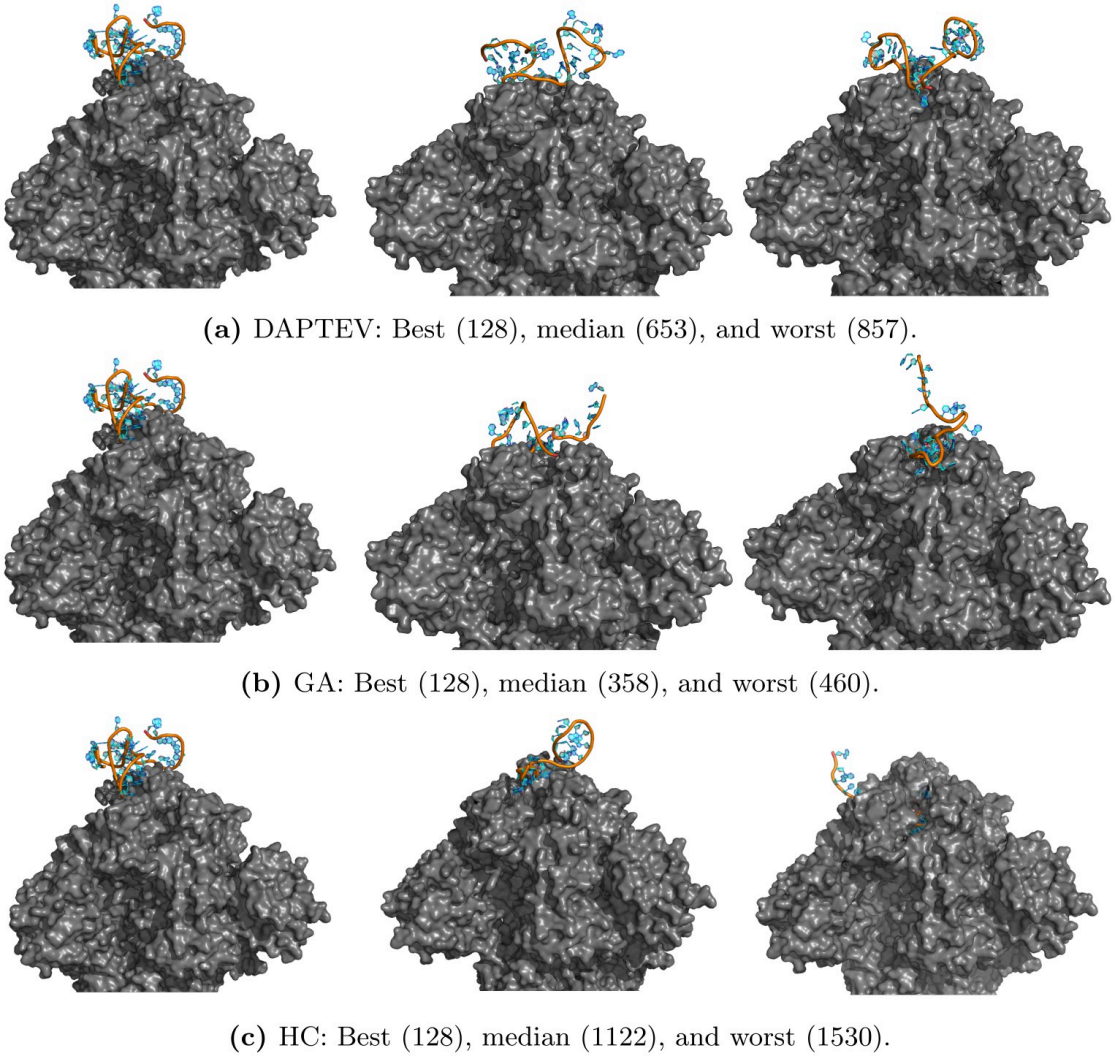
Folded RNAs docked to the SARS-CoV-2 RBD based on Rosetta-returned scores. Associated docking scores are in brackets next to the classification of each complex.

In Fig 8a, the best folded complex has a docking score of 128 with two base pairings in its secondary structure. Interestingly, every model produced the same best performing folded RNA. The median complex has a score of 653, also with two base pairings, and the worst has a score of 857 with one base pairing. Fig 8b has a median docking score of 358 with four base pairings, and the worst score of 460 has three base pairings. Every complex in Fig 8c has folded RNAs due to the nature of the dataset creation script. The median score is 1,122 with three base pairings and the worst score is 1,530 with one base pairing.

**Fig 9:**
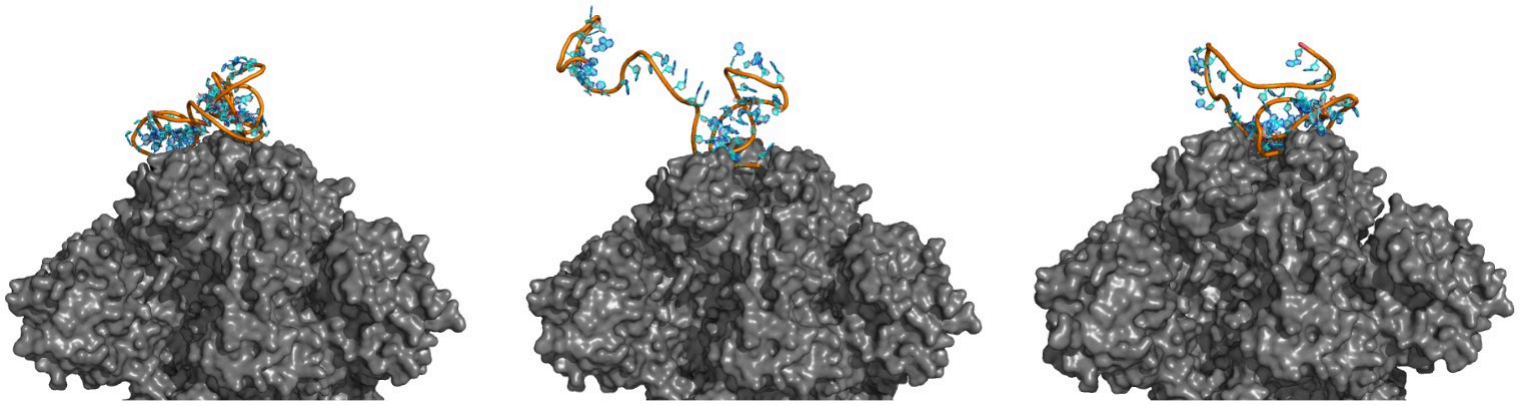
SARS-CoV-2 spike protein RBD targeting aptamers. CoV2-RBD-1C (left), CoV2–6C3 (middle), and nCoV-S1-Apt1 (right) docked to the cropped spike protein.

**Fig 10:**
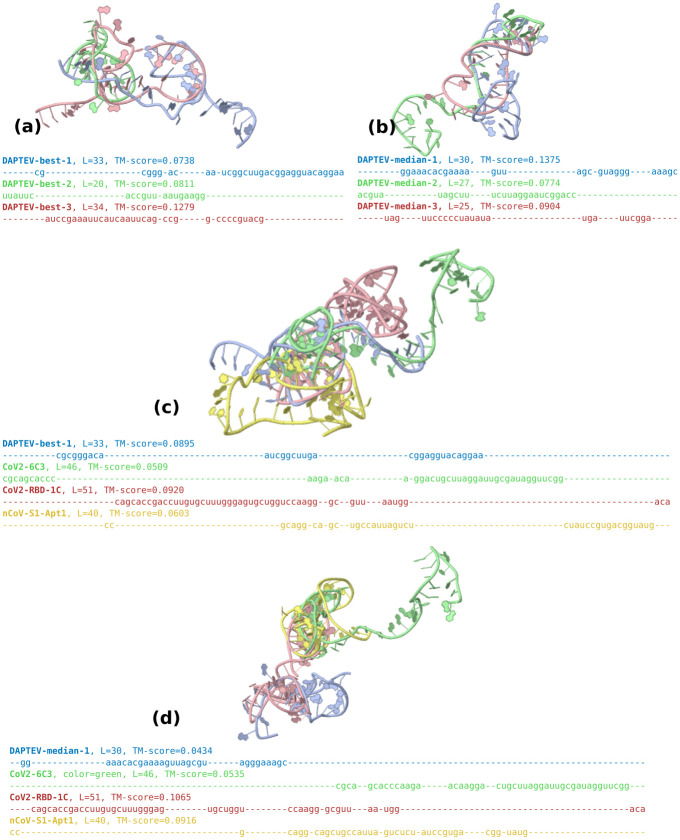
3D structure alignments of aptamers. (a) Alignment of the top three aptamers designed by DAPTEV. (b) Alignment of the median three aptamers designed by DAPTEV. (c) Alignment of the best aptamer designed by DAPTEV with three existing aptamers targeting the RBD of the SARS-CoV-2 spike protein. (d) Alignment of the median aptamer designed by DAPTEV with three existing aptamers targeting the RBD of the SARS-CoV-2 spike protein.

For a comparison, we found three recently published DNA SARS-CoV-2 spike protein RBD-targeting aptamers (CoV2-RBD-1C, CoV2–6C3, nCoV-S1-Apt1) (from Table 2 of [54]). Since our docking module only works for RNAs, we converted them to RNA via a base change of thymine to uracil, obtained their secondary structures via Arnie, and then ran them through the Rosetta docking program against our cropped version of the SARS-CoV-2 protein, constraining them to the RBD area. Fig 9 depicts the docked complexes. CoV2-RBD-1C is 51 nt long, has a docking score of 24,411, and contains 13 base pairs in its secondary structure. CoV2–6C3 is 46 nt long, has a docking score of 8,786, and contains 9 base pairs. nCoV-S1-Apt1 is 40 nt long, has a docking score of 20,799, and contains 9 base pairs. These three existing aptamers have much larger docking scores and more base pairs than the results obtained by DEPTEV. It is worth noting that the conversion to RNA may negatively affected the docking scores and increased the numbers of base pairs as RNA aptamers tend to form more enriched structures than DNA aptamers [54]. To compare these three real existing aptamers and the ones designed by DAPTEV in the 3D structure space, we applied the US-align platform [55] to compared the structure similarity among DAPTEV’s best three aptamers, among DAPTEV’s median three aptamers, between DAPTEV’s best and the three existing aptamers, and between DAPTEV’s median and the three existing aptamers. Fig 10 shows the comparison results including their associated TM-scores (ranging from 0 to 1 with 1 indicating an identical structure match, and ≥ 0.45 meaning the RNA structures share the same global topology). We can see that the top three (and median three) aptamers designed by DAPTEV are structurally diverse. Furthermore, DAPTEV’s best and median aptamers do not share significant topological similarity with the three existing aptamers.

### Validation using molecular dynamics

*Molecular dynamics* (MD) simulations were performed to validate the behaviour and characteristics of the generated aptamers. In particular, 8 aptamers were used in the MD simulations, comprising of the best (best-128, best-213, best-223), median (median-653, median-656, median-658), and worst (worst-53159, worst-60678) scoring sequences, where the numeric postfixes indicate the corresponding binding scores.

In preparation for MD simulation, the production of RNA-protein complex PDB files for each aptamer were generated using the Schrödinger PyMOL visualization package [56]. Afterwards, system assembly and MD production runs were conducted utilizing GROMACS [57]. Following the completed MD production runs, analysis and visualization were subsequently made using the in-built GROMACS utilities, alongside the *Visual Molecular Dynamics* (VMD) software package [58].

**Fig 11:**
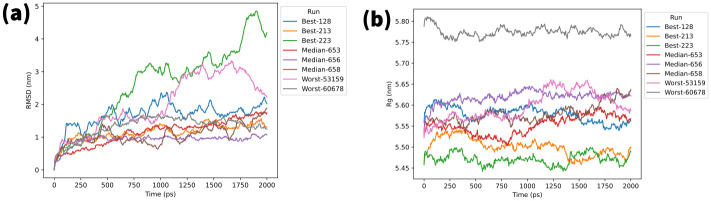
Molecular dynamics simulation results. (a) Root mean squared deviation (RMSD) of RNA aptamers, and (b) radius of gyration (Rg) for each RNA aptamer-protein complex. RMSD of the RNA aptamers was obtained after least square fit to the protein backbone.

To analyze the dynamics and stability of each RNA-protein complex, the *root mean squared deviation* (RMSD) for each aptamer was calculated with least-squares fitting to the protein backbone (Fig 11a). Measurement of RMSD allows for quantification of structural deviation over time, with reduced fluctuations and RMSD values potentially indicative of stability. Based on the MD simulation, apart from best-223 and worst-53159, each aptamer appears to have equilibrated by 500 ps. After 500 ps, the RMSD values had collectively ranged between 0.63 nm to 2.37 nm, suggesting aptamer stability with regards to low structural movement over time. Moreover, the standard deviation of each individual aptamer ranges from 0.04 nm to 0.28 nm, potentially indicative of aptamer stability regarding low structural fluctuation over time.

Furthermore, to observe the structural size and shape of the RNA-protein complex, the *radius of gyration* (Rg) was subsequently obtained for each RNA-protein complex following MD simulation (Fig 11b). Calculation of Rg values may be utilized in quantifying changes in structural compactness and stability of the RNA-protein complex over time. Based on the observed values after 500 ps, excluding worst-53159, Rg for each of the aptamer complexes remained stable with standard deviations of individual RNA-protein complex ranging between 0.008 nm to 0.025 nm. Therefore, in addition to the RMSD values, the Rd values may further indicate the stability of the generated aptamers based on low fluctuations in variation and compactness respectively. For more information on MD utilization, please see the S1 File supplementary document.

### Novelty and diversity of generated aptamers

Lastly, two additional metrics were calculated to assess model performance. These metrics, expressed as ratios, include novelty and diversity. *Novelty* is calculated as the number of generated sequences that do not exist in the initial training data versus the total number of generated sequences in a generation (the “population size”). *Diversity* is calculated as the number of generated unique sequences versus the total number of generated sequences in a generation. The novelty values calculated for DAPTEV and GA’s last generations are 0.8475 and 0.9962 respectively. Similarly, the diversity values were 0.8487 and 0.9937 respectively. Hence, both DAPTEV and GA show good novelty and diversity, and GA seems always to produce novel and unique sequences.

## Discussion and conclusion

### Observations

While it may seem that GA performed the best in terms of docking scores, this is not actually the case if the aptamer structures are considered as well. As previously mentioned, the docking score can be artificially improved by producing an unfolded RNA secondary structure which, in turn, will incur fewer penalties during the RNA tertiary structure prediction and the docking simulation. If a model prioritizes only unconnected structures, it stands to reason that it would seem to perform better when only considering score output. However, the expectation of this research is three-fold. (1) The docking scores has to be optimized. This was accomplished best by GA as substantiated by the returned scores and statistical analysis. (2) Some learning of well-performing structural motifs in the provided RNA secondary structures is required. Producing unfolded RNAs is not overly helpful when attempting to develop aptamer-based drugs. Even if this is a desirable trait, we expect to produce more complex structures from intelligent models and not be forced to sacrifice these features. Based on the percentage of folded structures in the last generation, it is clear that DAPTEV performs significantly better than GA. This points to the conclusion that GA solely prioritized the optimization of scores to the detriment of structural complexity. These results also indicate that DAPTEV is indeed able to learn structural motif patterns from the training data. (3) The deep learning model has to possess the ability to be queried for new, well-performing, structurally connected RNAs to explore aptamer-based drug discovery. This point implies the persistence of a trained model and a way to request new data. A GA must be trained every time new sequences are required. A VAE, conversely, can have its current state of learning saved and reloaded nearly immediately. Furthermore, new sequences can be obtained immediately. With everything mentioned above, one could posit that DAPTEV performs admirably in all three requirements. Additionally, every score produced in the last generation of DAPTEV was below the score threshold of 3,500, which was the docking score limit set initially to tell the VAE that a sequence was performing well. DAPTEV was able to learn structural features and other characteristics that make RNA perform well when docking to a specific target. Finally, the model can be saved and queried for new sequences that should perform well at docking to a specific target’s RBD while also maintaining some structural complexity. In contrast, GA and the hill climber algorithm must both be retrained every time, GA prioritized score optimization at the cost of motif preservation, and the hill climber is likely to yield poor results due to premature convergence at a local minimum.

### Limitations

There are some limitations of this research. Firstly, DAPTEV did not have enough starting aptamer data in the initial dataset. Only 849 unique, known, aptamer sequences were found during the literature review stage, but that was narrowed down further to only 344 due to parameter and computation time constraints.

Secondly, While this research was mainly focused on RNAs, some other research has worked on DNAs. It would have been preferable to include DNAs in the capabilities of this model. At present our RNA-protein docking module we are using may not be directly used for DNA aptamers because, in addition to the well-known difference nucleobases (U versus T), the sugars in RNAs and DNAs are also different. Due to these differences, RNA aptamers in general have more intrinsic structures than DNA aptamers. In our future work, we will develop an efficient DNA-protein docking module.

The randomly generated sequences must have at least one connection. However, more control over the secondary structure is preferred. The specification of connection amounts or a range of acceptable connections would be beneficial. Furthermore, the DAPTEV model is affected by limited accuracy from the structure/energy prediction process of both secondary and tertiary structures.

Currently, DAPTEV assumes that the latent space follows a Gaussian distribution. This distribution may not be robust enough to capture the complexity of the task. Testing different distributions could have yielded further insight into the VAE’s ability to operate in this problem space.

Several in silico designed aptamers were validated using MD simulation in this study. Even though MD is a convenient approach to gain insights into the interactions between aptamers and the target protein, it is limited with a simplified molecular environment model. Ideally, an experimental protocol should be established to synthesize these aptamers and validate their interactions with the spike protein.

### Conclusion

The goal of this research was to see if a deep generative model would be efficient at accelerating the RNA aptamer drug development process. While this research was applied to the SARS-CoV-2 spike protein, careful consideration was placed into the universal design for nearly any protein target. With regard to target affinity, one could conclude that both DAPTEV and GA performed well at this task. While the GA did outperform DAPTEV in this regard, the difference between these two models was not very large. Especially when considering that the score threshold was set to 3,500 and DAPTEV was still able to produce scores significantly lower than that. For fold rate, DAPTEV certainly shows some promising results. This is especially true when compared to GA. DAPTEV had an output with 34% of its produced sequences containing at least one connection in the secondary structure. This number could have been even higher if more vocabulary combinations had been implemented and some additional parameter tests were run. Conversely, GA only produced a 6% fold rate. This indicates that the VAE was indeed able to learn some structural features of the data and the MLP regularizer performed well at its task. The code for this model is accessible at https://github.com/candress/DAPTEV_Model.

### Future work

Several additional features and considerations could improve this research. (1) One such consideration is the fact that DAPTEV’s performance is improved when enriching the data with real aptamers. It would have been preferable to add more to the starting dataset. Unfortunately, at the time of searching, it seemed most of the aptamer datasets and databases were taken down. (2) Currently, this model only accepts RNA data. However, DNA aptamers are more common in literature. It would be beneficial to include the capability to work with DNA data. (3) Allowing the user to perform tertiary structure prediction separately, or to provide pre-calculated tertiary structure may be beneficial if a *prefold-then-fold-and-dock-then-refinement* strategy is implemented in the future. (4) Also, statically choosing the protein atom of CA and the RNA atom of C5 may be negatively affecting the performance as there is no determination of which atoms are more likely to interact. Instead, the interactions between each atom present in the protein residue and the possible RNA bases could have been pre-calculated. (5) It would be beneficial to increase the vocabulary combination amount. This DAPTEV only allows for combinations up to three characters long to reduce computation time. However, a vocabulary length of size three impedes the model’s ability to learn more complex structural motifs. (6) Some additional considerations and implementations could be the following: a learnable KL/reconstruction loss balancing component could be implemented so the model regulates the weights of the KL and the reconstruction loss [59]. Transformer is another versatile deep learning model that relies entirely on self-attention and is well-suited for design tasks [60], but requires more data to train. Given more data available in the future, it would be interesting to switch out the VAE with a Transformer to see how the results are affected. (7) The RNA-protein binding affinity score is the objective in our optimization process. In future development, other objectives, such as structural complexity and specificity via counter docking, will be considered. (8) It is difficult to make conclusive observations at this moment due to the few number of experimental runs. Ideally, at least 30 runs per experiment per model should be performed. However, this would take an inordinate amount of time given the current computing restrictions (Rosetta RNA-protein docking) for this research. Only way this would be feasible is if one had access to a cluster to distribute the workload. Another future option is to develop a GPU-empowered aptamer docking module which is expected to significantly improve the scalability of this research. (9) Our discovered new aptamers with promising binding affinity and folding rate to the SARS-CoV-2 spike protein can be tested in wet-lab experiments.

## Supporting information

S1 File Molecular dynamics methodology. More information regarding the method, considerations, and findings during the molecular dynamic simulations.
(PDF)

## References

[1] Kim TH, Lee SW. Aptamers for Anti-Viral Therapeutics and Diagnostics. International Journal of Molecular Sciences. 2021;22(8):4168. doi: 10.3390/ijms22084168 33920628 PMC8074132

[2] Lan J, Ge J, Yu J, Shan S, Zhou H, Fan S, et al. Structure of the SARS-COV-2 Spike Receptor-binding Domain Bound to the ACE2 Receptor. Nature. 2020;581(7807):215–220. doi: 10.1038/s41586-020-2180-5 32225176

[3] Song Y, Song J, Wei X, Huang M, Sun M, Zhu L, et al. Discovery of Aptamers Targeting the Receptor-Binding Domain of the SARS-CoV-2 Spike Glycoprotein. Analytical Chemistry. 2020;92(14):9895–9900. doi: 10.1021/acs.analchem.0c01394 32551560

[4] Tai W, He L, Zhang X, Pu J, Voronin D, Jiang S, et al. Characterization of the Receptor-binding Domain (RBD) of 2019 Novel Coronavirus: Implication for Development of RBD Protein as a Viral Attachment Inhibitor and Vaccine. Cellular and Molecular Immunology. 2020;17(6):613–620. doi: 10.1038/s41423-020-0400-4 32203189 PMC7091888

[5] Walls AC, Park YJ, Tortorici MA, Wall A, McGuire AT, Veesler D. Structure, Function, and Antigenicity of the SARS-CoV-2 Spike Glycoprotein. Cell. 2020;181(2):281–292.e6. doi: 10.1016/j.cell.2020.02.058 32155444 PMC7102599

[6] Yi C, Sun X, Ye J, Ding L, Liu M, Yang Z, et al. Key Residues of the Receptor Binding Motif in the Spike Protein of SARS-CoV-2 that Interact with ACE2 and Neutralizing Antibodies. Cellular & Molecular Immunology. 2020;17(6):621–630. doi: 10.1038/s41423-020-0458-z 32415260 PMC7227451

[7] Wengerter BC, Katakowski JA, Rosenberg JM, Park CG, Almo SC, Palliser D, et al. Aptamer-targeted Antigen Delivery. Molecular Therapy: The Journal of the American Society of Gene Therapy. 2014;22(7):1375–1387. doi: 10.1038/mt.2014.51 24682172 PMC4089008

[8] Yoon SY, Gee G, Hong KJ, Seo SH. Application of Aptamers for Assessment of Vaccine Efficacy. Clinical and Experimental Vaccine Research. 2017;6(2):160–163. doi: 10.7774/cevr.2017.6.2.160 28775981 PMC5540965

[9] Plotkin S, Robinson JM, Cunningham G, Iqbal R, Larsen S. The Complexity and Cost of Vaccine Manufacturing—An Overview. Vaccine. 2017;35(33):4064–4071. doi: 10.1016/j.vaccine.2017.06.003 28647170 PMC5518734

[10] Tuerk C, Gold L. Systematic Evolution of Ligands by Exponential Enrichment: RNA Ligands to Bacteriophage T4 DNA Polymerase. Science. 1990;249(4968):505–510. doi: 10.1126/science.2200121 2200121

[11] Kupakuwana GV, II JEC, McPike MP, Borer PN. Acyclic Identification of Aptamers for Human Alpha-thrombin Using Over-represented Libraries and Deep Sequencing. PloS One. 2020;6(5):e19395. doi: 10.1371/journal.pone.0019395 PMC3098231 21625587

[12] Ahirwar R, Nahar S, Aggarwal S, Ramachandran S, Maiti S, Nahar P. In Silico Selection Of An Aptamer To Estrogen Receptor Alpha Using Computational Docking Employing Estrogen Response Elements As Aptamer-Alike Molecules. Scientific Reports. 2016;6(1):21285. doi: 10.1038/srep21285 26899418 PMC4761961

[13] Lee G, Jang GH, Kang HY, Song G. Predicting Aptamer Sequences that Interact with Target Proteins Using an Aptamer-protein Interaction Classifier and a Monte Carlo Tree Search Approach. PLOS ONE. 2021;16(6):e0253760. doi: 10.1371/journal.pone.0253760 34170922 PMC8232527

[14] Song J, Zheng Y, Huang M, Wu L, Wang W, Zhu Z, et al. A Sequential Multidimensional Analysis Algorithm for Aptamer Identification Based on Structure Analysis and Machine Learning. Analytical Chemistry. 2020;92(4):3307–3314. doi: 10.1021/acs.analchem.9b05203 31876151

[15] Becker KCD, Becker RC. Nucleic Acid Aptamers as Adjuncts to Vaccine Development. Current Opinion in Molecular Therapeutics. 2006;8(2):122–129. 16610764

[16] Chen Z, Hu L, Zhang BT, Lu A, Wang Y, Yu Y, et al. Artificial Intelligence in Aptamer–Target Binding Prediction. International Journal of Molecular Sciences. 2021;22(7):3605. doi: 10.3390/ijms22073605 33808496 PMC8038094

[17] Keefe AD, Pai S, Ellington A. Aptamers as Therapeutics. Nature Reviews Drug Discovery. 2010;9(7):537–550. doi: 10.1038/nrd3141 20592747 PMC7097324

[18] Kinghorn AB, Fraser LA, Lang S, Shiu SCC, Tanner JA. Aptamer Bioinformatics. International Journal of Molecular Sciences. 2017;18(12). doi: 10.3390/ijms18122516 29186809 PMC5751119

[19] Wornow M. Applying Deep Learning to Discover Highly Functionalized Nucleic Acid Polymers That Bind to Small Molecules [Bachelor’s Thesis]. Harvard College. Cambridge, USA; 2020.

[20] Zou X, Wu J, Gu J, Shen L, Mao L. Application of Aptamers in Virus Detection and Antiviral Therapy. Frontiers in Microbiology. 2019;10:1462. doi: 10.3389/fmicb.2019.01462 31333603 PMC6618307

[21] Russell S, Norvig P. Artificial Intelligence: A Modern Approach. 4th ed. Pearson; 2020.

[22] Bishop CM. Pattern Recognition and Machine Learning. New York: Springer; 2006.

[23] LeCun Y, Bengio Y, Hinton G. Deep Learning. Nature. 2015;521:436–444. doi: 10.1038/nature14539 26017442

[24] Romez-Bombarelli R, Wei JN, Duvenaud D, Hernarndez-Lobato JM, Sanchez-Lengeling B, Sheberla D, et al. Automatic Chemical Design Using a Data-driven Continuous Representation of Molecules. ACS Central Science. 2018;4:268–276. doi: 10.1021/acscentsci.7b00572 29532027 PMC5833007

[25] Kim J, Park S, Min D, Kim W. Comprehensive Survey of Recent Drug Discovery Using Deep Learning. International Journal of Molecular Sciences. 2021;22:9983. doi: 10.3390/ijms22189983 34576146 PMC8470987

[26] Grantham K, Mukaidaisi M, Ooi HK, Ghaemi MS, Tchagang A, Li Y. Deep Evolutionary Learning for Molecular Design. IEEE Computational Intelligence Magazine. 2022;17(2):14–28. doi: 10.1109/MCI.2022.3155308

[27] Mukaidaisi M, Vu A, Grantham K, Tchagang A, Li Y. Multi-objective Drug Design Based on Graph-fragment Molecular Representation and Deep Evolutionary Learning. Frontier in Pharmacology. 2022;13:920747. doi: 10.3389/fphar.2022.920747 35860028 PMC9291509

[28] Jiang P, Meyer S, Hou Z, Propson NE, Soh HT, Thomson JA, et al. MPBind: A Meta-motif-based Statistical Framework and Pipeline to Predict Binding Potential of SELEX-derived Aptamers. Bioinformatics. 2014;30(18):2665–2667. doi: 10.1093/bioinformatics/btu348 24872422 PMC4155251

[29] Hoinka J, Berezhnoy A, Sauna ZE, Gilboa E, Przytycka TM. AptaCluster—A Method to Cluster HT-SELEX Aptamer Pools and Lessons from Its Application. In: International Conference on Research in Computational Molecular Biology; 2014. p. 115–128. 10.1007/978-3-319-05269-4_9 PMC4281958 25558474

[30] Alam KK, Chang JL, Burke DH. FASTAptamer: A Bioinformatic Toolkit for High-throughput Sequence Analysis of Combinatorial Selections. Molecular Therapy—Nucleic Acids. 2015;4:e230. doi: 10.1038/mtna.2015.4 25734917 PMC4354339

[31] Hiller M, Pudimat R, Busch A, Backofen R. Using RNA Secondary Structures to Guide Sequence Motif Finding Towards Single-stranded Regions. Nucleic Acids Research. 2006;34(17):e117. doi: 10.1093/nar/gkl544 16987907 PMC1903381

[32] Hoinka J, Zotenko E, Friedman A, Sauna ZE, Przytycka TM. Identification of Sequence-structure RNA Binding Motifs for SELEX-derived Aptamers. Bioinformatics. 2012;28(12):i215–i223. doi: 10.1093/bioinformatics/bts210 22689764 PMC3371842

[33] Dao P, Hoinka J, Takahashi M, Zhou J, Ho M, Wang Y, et al. AptaTRACE Elucidates RNA Sequence-Structure Motifs from Selection Trends in HT-SELEX Experiments. Cell Systems. 2016;3:62–70. doi: 10.1016/j.cels.2016.07.003 27467247 PMC5042215

[34] Li BQ, Zhang YC, Huang GH, Cui WR, Zhang N, Cai YD. Prediction of Aptamer-Target Interacting Pairs with Pseudo-Amino Acid Composition. PLoS ONE. 2014;9(1):e86729. doi: 10.1371/journal.pone.0086729 24466214 PMC3899287

[35] Zhang L, Zhang C, Gao R, Yang R, Song Q. Prediction of Aptamer-protein Interacting Pairs Using an Ensemble Classifier in Combination with Various Protein Sequence Attributes. BMC Bioinformatics. 2016;17:225. doi: 10.1186/s12859-016-1087-5 27245069 PMC4888498

[36] Peng C, Han S, Zhang H, Li Y. RPITER: A Hierarchical Deep Learning Framework for ncRNA–Protein Interaction Prediction. International Journal of Molecular Sciences. 2019;20:1070. doi: 10.3390/ijms20051070 30832218 PMC6429152

[37] Im J, Park B, Han K. A Generative Model for Constructing Nucleic Acid Sequences Binding to a Protein. BMC Genomics. 2019;20(13):967. doi: 10.1186/s12864-019-6299-4 31881936 PMC6933682

[38] Alipanhi B, Delong A, Weirauch MT, Frey BJ. Predicting the Sequence Specificities of DNA- and RNA-binding Proteins by Deep Learning. Nature Biotechnology. 2015;33(8):831–838. doi: 10.1038/nbt.3300 26213851

[39] Park B, Han K. Discovering Protein-binding RNA Motifs with a Generative Model of RNA Sequences. Computational Biology and Chemistry. 2020;84. doi: 10.1016/j.compbiolchem.2019.107171 31931434

[40] Iwano N, Adachi T, Aoki K, Nakamura Y, Hamada M. RaptGen: A Variational Autoencoder with Profile Hidden Markov Model for Generative Aptamer Discovery. bioRxiv. 2021; p. 2021.02.17.431338.

[41] Chaslot GMJB, Winands MHM, Herik HJVD, Uiterwijk JWHM. Progressive Strategies for Monte-Carlo Tree Search. New Mathematics and Natural Computation. 2008;4(3):343–357. doi: 10.1142/S1793005708001094

[42] Kappel K, Das R. Sampling Native-like Structures of RNA-Protein Complexes Through Rosetta Folding and Docking. Structure. 2019;27(1):140–151.e5. doi: 10.1016/j.str.2018.10.001 30416038 PMC6318048

[43] Wayment-Steele HK, Kladwang W, Strom AI, Lee J, Treuille A, Das R. RNA Secondary Structure Packages Evaluated and Improved by High-throughput Experiments. bioRxiv. 2021;. 10.1038/s41592-022-01605-0 PMC9839360 36192461

[44] Kingma DP, Welling M. Auto-Encoding Variational Bayes. In: International Conference on Learning Representations; 2014.

[45] Chung J, Gulcehre C, Cho K, Bengio Y. Empirical Evaluation of Gated Recurrent Neural Networks on Sequence Modeling. In: NIPS 2014 Deep Learning and Representation Learning Workshop; 2014.

[46] Mikolov T, Chen K, Corrado GS, Dean J. Efficient Estimation of Word Representations in Vector Space. In: International Conference on Learning Representations; 2013.

[47] Pennington J, Socher R, Manning C. GloVe: Global Vectors for Word Representation. In: Conference on Empirical Methods in Natural Language. Doha, Qatar: Association for Computational Linguistics; 2014. p. 1532–1543.

[48] Fu H, Li C, Liu X, Gao J, Celikyilmaz A, Carin L. Cyclical Annealing Schedule: A Simple Approach to Mitigating KL Vanishing. ArXiv. 2019; p. arXiv.1903.10145.

[49] Premkumar L, Segovia-Chumbez B, Jadi R, Martinez DR, Raut R, Markmann A, et al. The Receptor Binding Domain of the Viral Spike Protein is an Immunodominant and Highly Specific Target of Antibodies in SARS-CoV-2 Patients. Science Immunology. 2020;5(48):eabc8413. doi: 10.1126/sciimmunol.abc8413 32527802 PMC7292505

[50] Markley JL, Bax A, Arata Y, Hilbers CW, Kaptein R, Sykes BD, et al. Recommendations for the Presentation of NMR Structures of Proteins and Nucleic Acids—IUPAC-IUBMB-IUPAB Inter-Union Task Group on the Standardization of Data Bases of Protein and Nucleic Acid Structures Determined by NMR Spectroscopy. Journal of Biomolecular NMR. 1998;12(1):1–23. doi: 10.1023/A:1008290618449 9729785

[51] Eaton BE, Gold L, Zichi DA. Let’s Get Specific: The Relationship Between Specificity and Affinity. Chemistry & Biology. 1995;2(10):633–638. doi: 10.1016/1074-5521(95)90023-3 9383468

[52] Berman HM, Westbrook J, Feng Z, Gilliland G, Bhat TN, Weissig H, et al. The Protein Data Bank. Nucleic Acids Research. 2000;28(1):235–242. doi: 10.1093/nar/28.1.235 10592235 PMC102472

[53] Li BQ, Zhang YC, Huang GH, Cui WR, Zhang N, Cai YD. Prediction of Aptamer-Target Interacting Pairs with Pseudo-Amino Acid Composition. PLOS ONE. 2014;9(1):e86729. doi: 10.1371/journal.pone.0086729 24466214 PMC3899287

[54] Zhang Y, Juhas M, Kwok CK. Aptamers Targeting SARS-COV-2: a Promising Tool to Fight Against COVID-19. Trends in Biotechnology. 2023;41(4):528–544. doi: 10.1016/j.tibtech.2022.07.012 35995601 PMC9340053

[55] Zhang C, Shine M, Pyle AM, Marie A, Zhang Y. US-align: Universal Structure Alignments of Proteins, Nucleic acids, and Macromolecular Complexes. Nature Methods. 2022;19:1109–1115. doi: 10.1038/s41592-022-01585-1 36038728

[56] Schrödinger L, DeLano W. The PyMOL Molecular Graphics System, Version 1.8; 2015.

[57] Berendsen HJC, van der Spoel D, van Drunen R. GROMACS: A Message-passing Parallel Molecular Dynamics Implementation. Computer Physics Communications. 1995;91(1):43–56. doi: 10.1016/0010-4655(95)00042-E

[58] Humphrey W, Dalke A, Schulten K. VMD—Visual Molecular Dynamics. Journal of Molecular Graphics. 1996;14:33–38. doi: 10.1016/0263-7855(96)00018-5 8744570

[59] Asperti A, Trentin M. Balancing Reconstruction Error and Kullback-Leibler Divergence in Variational Autoencoders. arXiv. 2020; p. ArXiv.2002.07514.

[60] Vaswani A, Shazeer N, Parmar N, Uszkoreit J, Jones L, Gomez AN, et al. Attention is All You Need. In: Neural Information Processing Systems; 2017.

