# Supplementary material for: DAPTEV: Deep aptamer evolutionary modelling for COVID-19 drug design
Source: PLoS Comput Biol. 2023 Jul 5;19(7):e1010774. doi: 10.1371/journal.pcbi.1010774 (PMC10351721; doi:10.1371/journal.pcbi.1010774)
Supplement: S1 File — More information regarding the method, considerations, and findings during the molecular dynamic simulations. (PDF) [file pcbi.1010774.s001.pdf]

Supplementary File
Molecular Dynamics Methodology

Aptamer Selection

Molecular dynamics (MD) simulations were utilized for the characterization of the generated aptamers, and ultimately, the validation of experimental results. To comparatively assess MD simulation, 9 aptamers were initially chosen for MD simulation, comprising of the three best (best-128, best-213, best 223), three median (median-653, median-656, median-658), and three worst (worst-53159, worst-53600, worst-60678) scoring sequences. The aptamers were named based on their group, with numeric postfixes indicating the corresponding binding scores.

PDB Preparation

The Schrödinger PyMOL visualization package [1] was utilized for the preparation of RNA-protein PDB files for each aptamer, with intention to perform MD simulations with GROMACS [2]. Using PyMOL, the docked PDF files of each aptamer were bound to the whole SARS-CoV-2 spike glycoprotein (closed state, PDB: 6VXX) to prevent instability during the MD simulation production runs. Moreover, the original aptamer PDB files were unable to processed natively by GROMACS, and thus, were modified in PyMOL (in particular, the addition of RNA terminal O5' groups) for compatibility with GROMACS topology.

Topology Preparation, Solvation, and Neutralization

The environment was prepared for MD simulation using various GROMACS utilities (*gmx*). Through *gmx pdb2gmx*, the *tip3p* water model was utilized alongside the *amber03* forcefield model. Hydrogens were also allowed to be handled and reconstructed by *gmx pdb2gmx*. The simulation size was defined by *gmx editconf*, where the molecules had been centered and positioned at least 1.0 nm from within the dodecahedron box edge. Solvation of the simulation box was performed utilizing *gmx solvate* with the *spc216.gro* pre-equilibrated water box. Additionally, the system was neutralized by the addition sodium and chlorine ions through *gmx genion*.

Energy Minimization and Equilibration

In preparation for MD simulation, each RNA-protein complex was put through energy minimization and environmental equilibration using *gmx grompp* and *gmx mdrun* to establish standard conditions for simulation. Energy had been minimized until values of less than 1000.0 kJ/mol/nm were established (Fig Aa). Notably, 8 out of 9 aptamer RNA-protein complexes were able to successfully undergo energy minimization; bottom-53600 was unable to be minimized, potentially due to the presence of overlapping atoms. This may be suggested as a limitation potentially stemming from inaccuracies in the Rosetta docking method in atomic position determination [3]. Of the remaining aptamers, each had successfully undergone equilibration with both *constant Number of particles, Volume, and Temperature* (NVT), alongside with *constant Number of particles, Pressure, and Temperature* (NPT). NVT equilibration had been set at a constant 300°K (Fig Ab), followed by NPT equilibration with an additional restraint of 1 bar (Fig Ac).

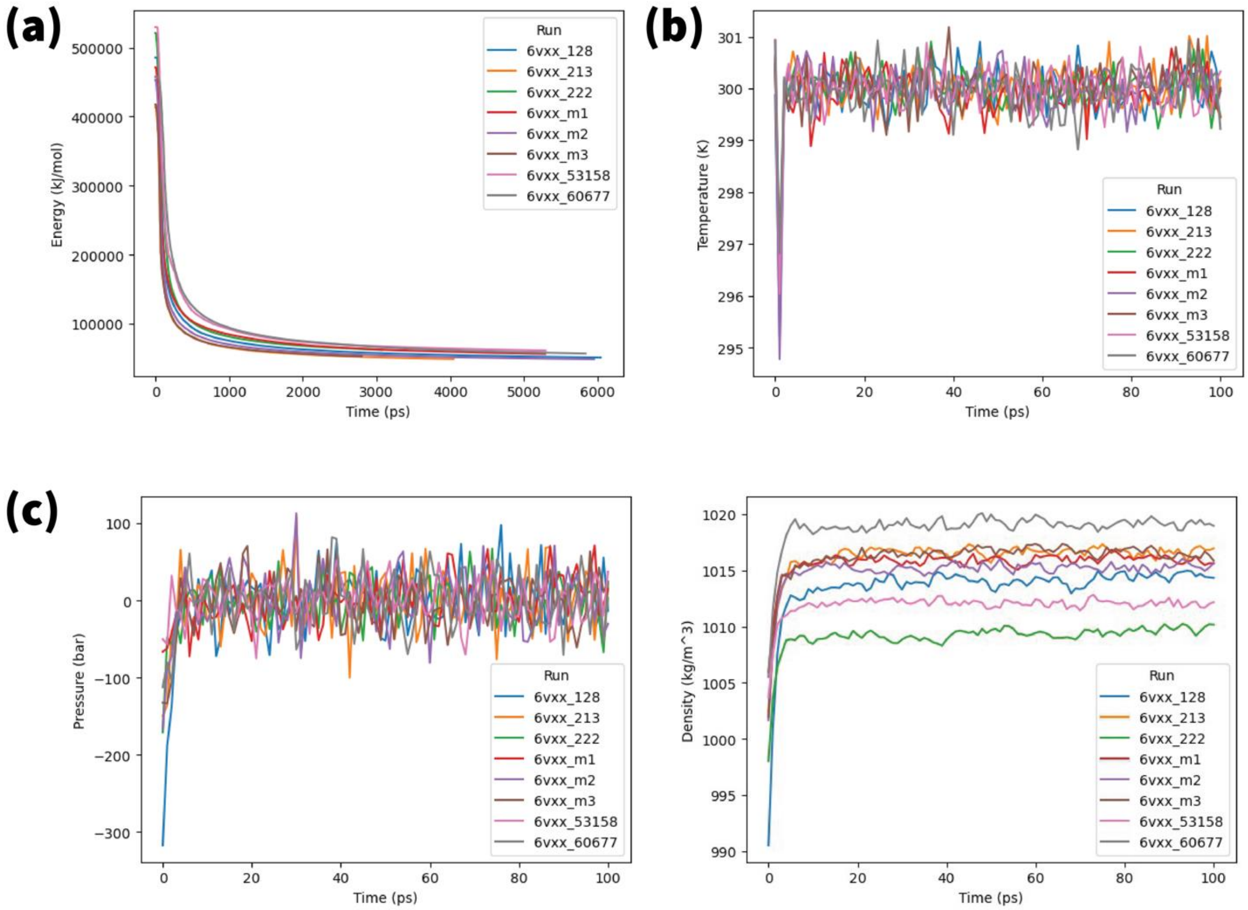

Fig A. Energy minimization and equilibration results. **(a):** Run energy during energy minimization step for MD simulation preparation. Energy had been minimized until values of less than 1000.0 kJ/mol/nm were established. **(b):** Run temperature during NVT equilibration step for MD simulation preparation. Temperature had been set to equilibrate at a constant 300°K. **(c):** Run pressure and density during NPT equilibration step for MD simulation preparation. Pressure had been set to equilibrate at a constant 1 bar.

MD Production Run and Analysis

Following equilibration, each RNA-protein complex had undergone an MD simulation production run utilizing *gmx grompp* and *gmx mdrun*. Production runs were ran for 1 000 000 steps, at 0.002 ps per step, resulting in an overall simulation length of 2000 ps. Energy and temperature were saved every 1.0 ps. Resulting simulation trajectory files were first processed utilizing *gmx trjconv*, prior to analysis using *gmx rms* and *gmx gyrate* for root mean squared deviation (RMSD) and radius of gyration calculations respectively. Visual analysis and assessment was further completed utilizing Visual Molecular Dynamics (VMD) software [4].

References

1. Schrödinger L, DeLano W. The PyMOL Molecular Graphics System, Version 1.8; 2015.
2. Berendsen HJC, van der Spoel D, Van Drunen R. GROMACS: A Message-passing Parallel Molecular Dynamics Implementation. Computer Physics Communications. 1995;91(1):43-56. doi:https://doi.org/10.1016/0010-4655(95)00042-E.
3. Kappel K, Das R. Sampling Native-like Structures of RNA-Protein Complexes Through Rosetta Folding and Docking. Structure. 2019;27(1):140-151.e5.
4. Humphrey W, Dalke A, Schulten K. VMD – Visual Molecular Dynamics. Journal of Molecular Graphics. 1996;14:33-38.
